# Identification and characterization of jasmonic acid- and linolenic acid-mediated transcriptional regulation of secondary laticifer differentiation in *Hevea brasiliensis*

**DOI:** 10.1038/s41598-019-50800-1

**Published:** 2019-10-04

**Authors:** Swee Cheng Loh, Ahmad Sofiman Othman, G. Veera Singham

**Affiliations:** 1 Centre for Chemical Biology, Universiti Sains Malaysia, 10 Persiaran Bukit Jambul, 11900 Bayan Lepas, Penang, Malaysia; 2 School of Biological Sciences, Universiti Sains Malaysia, 11800 Penang, Malaysia

**Keywords:** Plant development, Plant molecular biology, Plant physiology

## Abstract

*Hevea brasiliensis* remains the primary crop commercially exploited to obtain latex, which is produced from the articulated secondary laticifer. Here, we described the transcriptional events related to jasmonic acid (JA)- and linolenic acid (LA)-induced secondary laticifer differentiation (SLD) in *H. brasiliensis* clone RRIM 600 based on RNA-seq approach. Histochemical approach proved that JA- and LA-treated samples resulted in SLD in *H. brasiliensis* when compared to ethephon and untreated control. RNA-seq data resulted in 86,614 unigenes, of which 2,664 genes were differentially expressed in JA and LA-induced secondary laticifer harvested from *H. brasiliensis* bark samples. Among these, 450 genes were unique to JA and LA as they were not differentially expressed in ethephon-treated samples compared with the untreated samples. Most transcription factors from the JA- and LA-specific dataset were classified under MYB, APETALA2/ethylene response factor (AP2/ERF), and basic-helix-loop-helix (bHLH) gene families that were involved in tissue developmental pathways, and we proposed that Bel5-GA2 oxidase 1-KNOTTED-like homeobox complex are likely involved in JA- and LA-induced SLD in *H. brasiliensis*. We also discovered alternative spliced transcripts, putative novel transcripts, and *cis*-natural antisense transcript pairs related to SLD event. This study has advanced understanding on the transcriptional regulatory network of SLD in *H. brasiliensis*.

## Introduction

Laticifers are highly specialized cells or vessels that exist in approximately 12,500 plant species from more than 20 families^1–3^. Of these, only approximately 2,500 laticiferous plant species biosynthesize rubber and only a few biosynthesize high molecular weight rubber for commercial use^4^: rubber trees (*Hevea brasiliensis*), guayule (*Parthenium argentatum Gray*), and dandelions (*Taraxacum kok-saghyz*)^5^. Among these species, *H. brasiliensis* is the main source of industrial rubber due to its high yield, superior qualities, easy harvesting, and processing. Laticifers are firstly described by H. A. de Bary by their origin, development, and overall anatomy but not by their content^6^. Laticifers can be classified into articulated or non-articulated laticifer based on its origin^3,6^. Non-articulated type can be further subdivided into branched and unbranched whereas articulated type can be subcategorised into anastomosing and non-anastomosing^3^. Both are tubular structure and therefore it is also known as latex vessels. Both articulated and non-articulated types of laticifer are present in *H. brasiliensis*^7^. In *H. brasiliensis*, non-articulated laticifer presents in the primary phloem which can be found in the young organs in the primary state of growth whereas articulated laticifer presents in the secondary phloem distributed in the bark^8–11^. Unbranched, non-articulated laticifer is likely to differentiate in developing leaves or beneath apical meristems and elongate as the plant grows. Branched, non-articulated laticifer develops from few embryonic initials and profusely bifurcated and elongated forming a highly branched network throughout the plant^12,13^. The differentiation of articulated laticifer is coordinated with the development of other phloem tissues, with the initials forming longitudinal rings. It is distributed from the base to the top of the trunk with its density showed clonal variation^14,15^. Perforation of end walls occurs in most species and leads to the formation of continuous, multinucleate cytoplasm. Anastomoses form between adjacent articulated laticifers through cell wall degradation. Nevertheless, the successive differentiated laticifers from the cambium are organized in non-interconnected cylindrical rings^3,11^.

The latex that is commercially exploited to obtain rubber comes from the articulated secondary laticifer in *H. brasiliensis*^8–10^. It has been reported that the number of laticifer is one of the most important characteristics that affect the latex yield^11^. Therefore, inducing the differentiation of more secondary laticifers is a promising method to improve the latex yield^16^. Articulated laticifer is genetically controlled, may be induced by the signal of ageing primary laticifer, or possibly influenced by environmental stresses, which are yet to be determined^17–19^. Besides, wounding which is often done during commercial exploitation, is an important factor that induces laticifer ring formation^20,21^. In addition, the application of exogenous jasmonic acid (JA), methyl jasmonate, linolenic acid (LA), hydrogen peroxide, polyethylene glycol 6000, trichostatin A, or coronatine could induce the secondary laticifer differentiation (SLD) in *H. brasiliensis* but this is not the case for ethylene, salicylic acid or abscisic acid treatment^18,22–24^. Among the factors that could induce SLD, JA is a phytohormone where its signalling pathway has been studied extensively^25,26^. LA is the precursor of JA and therefore they share a similar pathway^27^.

Although there has been great progress in investigating the distinct loci (LOT OF LATEX, LOL) regulating the latex production in the non-articulated laticifer of JA-treated *Euphorbia lathyris*^12,28^, little is known about the SLD within the secondary phloem^29–31^. In our previous microarray-based gene expression profiling study, which analysed 27,195 gene models, genes and signalling pathways that are possibly associated with JA- and LA-induced SLD were identified^32^. However, the complex signalling networks using 39.44% of total identified 68,955 gene models from the first *Hevea* draft genome^33^ are less well-understood. Moreover, transcriptional events were masked as the exact transcript structures could not be identified via microarray analysis^32^. Thus, we performed in-depth and high-throughput strand-specific RNA-sequencing, ssRNA-seq to investigate the transcriptional events behind the SLD in *H. brasiliensis* clone RRIM 600. By applying this approach, we conducted differential gene expression analyses on untreated *Hevea* samples (CTRL), and JA-, LA-, and ethephon (ET)-treated *Hevea* bark samples by incorporating the recently published *Hevea* draft genome as reference^34^. Moreover, we uncovered alternative splicing (AS) events, *cis*-natural antisense transcripts, and novel transcripts that possibly occur following JA- and LA-induced SLD. We also investigated the effect of JA and LA in inducing SLD through histochemical approach. Subsequently, we investigated the relative expression of a set of randomly selected transcription factors (TFs) that are differentially expressed in JA- and LA- treated bark samples among different JA-treated *Hevea* clones namely RRIM 600, RRIM 2025, and RRIM 3001, to observe the consistency of the expression pattern of the selected TFs across the different *Hevea* clones. A consistent expression pattern across the different JA-treated clones would validate the involvement of the tested TFs in SLD regulatory network. This study provides a useful platform for future research to further investigate the functional role of the key regulatory genes that regulate the SLD and enhance the molecular breeding of *H. brasiliensis* clones with an increased number of laticifers.

## Results and Discussion

### Secondary laticifer induction in Hevea bark tissue

**Figure 1:**
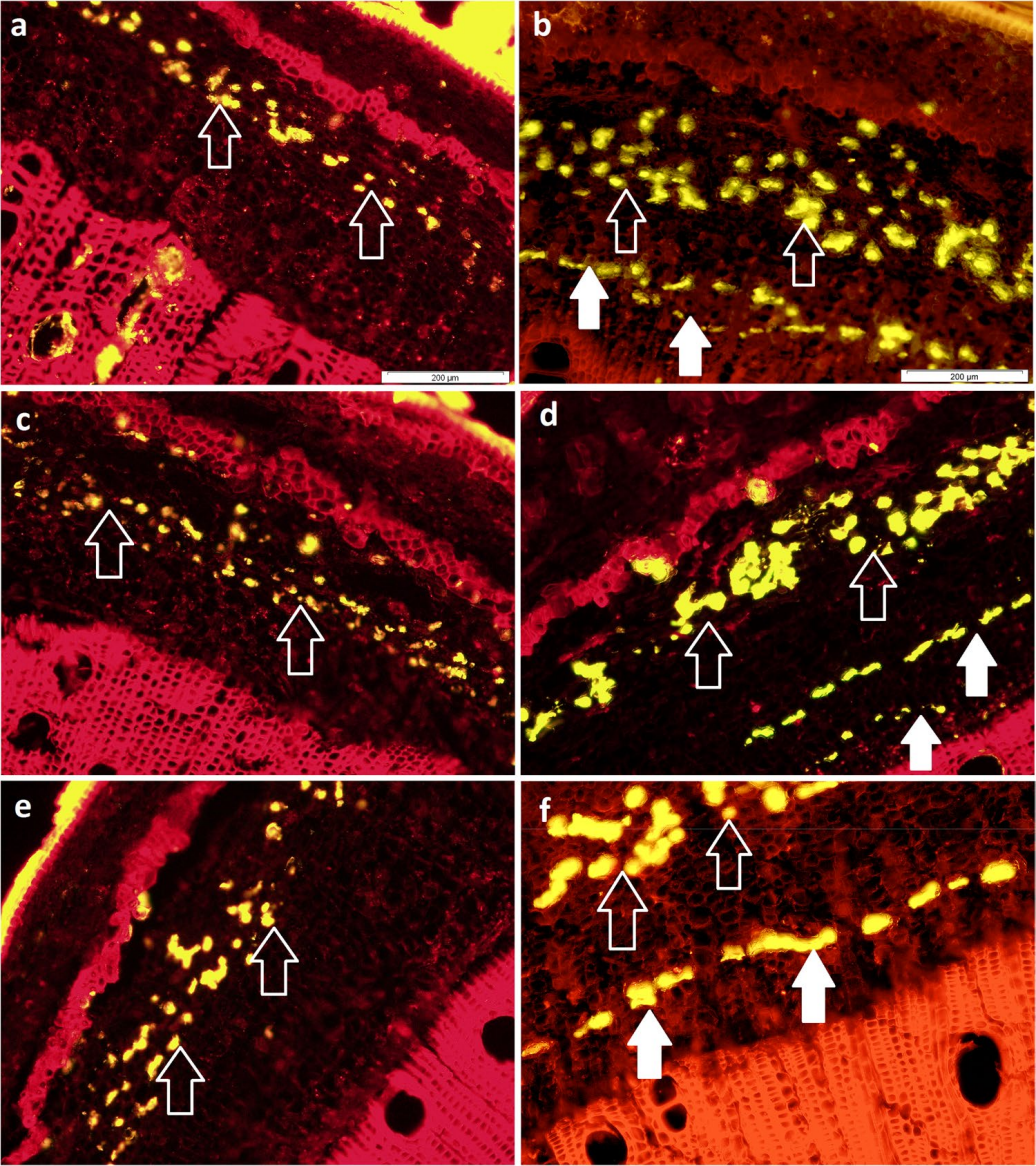
Comparison of transverse sections of *H. brasiliensis* bark samples in JA-treated *Hevea* clones after 60 days of treatment (right panels) and untreated *Hevea* clones (left panels). (**a**) Untreated RRIM 600 (**b**) JA-treated RRIM 600 (**c**) untreated RRIM 2025 (**d**) JA-treated RRIM 2025 (**e**) untreated RRIM 3001 (**f**) JA-treated RRIM 3001. White outlined arrows indicate primary laticifer (PL) while white arrows indicate induced secondary laticifer (SL). Scale bar: 200 µm.

The number of laticifer is one of the most important factors that affects the latex yield^11^. In our study, one layer of secondary laticifer was successfully induced by JA and LA treatments respectively in the bark samples of RRIM 600. A new ring formation can be seen in Fig. 1b,d, and Supplementary Fig. S1c indicating that there is a developmental effect by JA and LA treatment after two months. This is in line with a study where the selected gene expression levels in methyl jasmonate-treated RRIM 600 and RRIT 251 is still significantly different compared to control up to 3 months^35^. However, no secondary laticifer was observed in the CTRL or ET-treated plants of the same age (Supplementary Fig. S1). This observation is in line with other scientific reports^18,32^.

**Figure 2:**
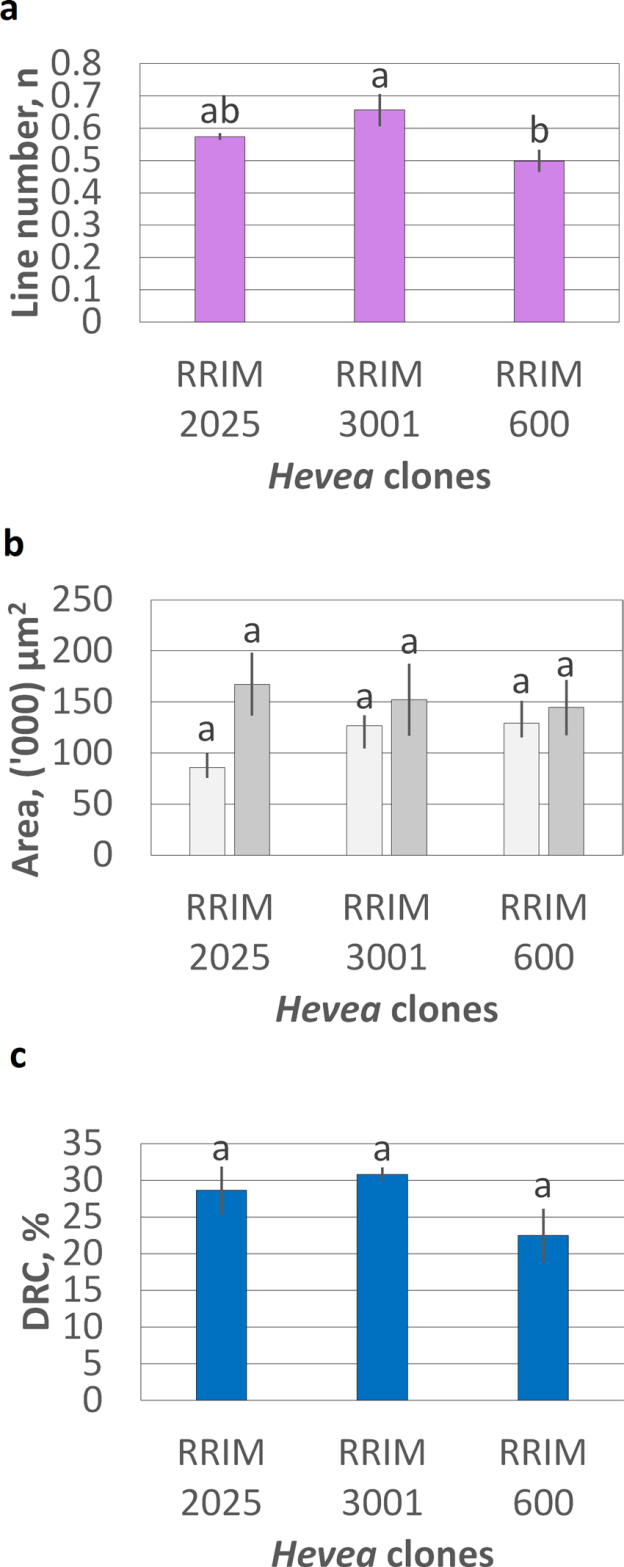
The quantification of laticifers and DRC content in JA-treated *Hevea* clones after 60 days of treatment and untreated *Hevea* clones. (**a**) The JA-induced secondary laticifer line number in 1 mm distance. There is no secondary laticifer in the untreated clones (Fig. 1a,c,e). (**b**) The total mean area (µm^2^) per mm^2^ region of the primary laticifer distribution within untreated and JA-treated clones is represented by light grey bars and dark grey bars respectively. (**c**) The percentage of DRC over total collected latex in JA-treated clones. Statistical tests among the *Hevea* clones were performed through a one-way ANOVA, followed by Tukey’s post-hoc test at a significance level of 0.05. Each vertical line on the bar represents mean ± SEM across the biological replicates. Mean values with different alphabets are significantly different.

Secondary laticifer was also observed in RRIM 2025 and RRIM 3001 following JA treatment. However, the effect of JA treatment on different clones showed different extent of induced secondary laticifer line number (Fig. 1). The JA-induced secondary laticifer line number was the highest in the clone RRIM 3001 (Fig. 2a). This could have been due to its’ rapid growth rate and high latex-yielding properties^36,37^. In addition to SLD, exogenous JA could induce more primary laticifer numbers in *Hevea*^18^. However, there was no significant difference in the primary laticifer distribution among the JA-treated clones. In addition to laticifer quantification, an important parameter (dry rubber content, DRC) for the purpose of trading in the rubber industry was determined in the present study. Similar to the primary laticifer distribution, the DRC was not significantly different among these clones (Fig. 2b,c). This is the first report that describes clonal variation in the JA-induced secondary laticifer line numbers in *Hevea*, suggesting differential effect of clonal variety in response to phytohormones such as JA.

### Summary statistics of the ssRNA-seq data

The total raw reads of the eight sequencing samples derived from *Hevea* bark samples of RRIM 600 ranged from 93.6 to 103.7 million (Supplementary Table S1). In addition to achieving a total of 364,900,713 high quality reads, more than 84.07% of the total clean reads (Table 1) were aligned to the recently published rubber draft genome^34^ generating 86,614 unigenes that were identified from 161,652 transcripts. This is more than the predicted 68,955 gene models^33^, 67,873 unigenes^38^, and 54,689 unigenes^39^ from other studies. Therefore, the assembly data in the present study extend the rubber tree bark transcriptome database and will help reveal the regulatory networks involved in the SLD from the bark region in RRIM 600. In the present study, the retained strand information of the assembled transcripts disclosed that approximately half of the total genes were transcribed from each DNA strand in all samples (Supplementary Table S2). Furthermore, we discovered 10,242 overlapping genes transcribed from both strands at 4,495 genomic locations, suggesting possible *cis*-natural antisense transcript pairs at these locations. These genes might participate in self-regulatory circuits that allow them to regulate their own expression^40^ (Supplementary Table S3).

**Table 1:** The total number of quality reads, the rate of overall reads mapping, and the rate of aligned reads across the samples.

Sample	Raw sequenced paired end reads	Quality reads	Overall reads mapping (%)	Aligned reads (%)
CTRL_0	51,827,476	47,961,543 (92.54%)	87.55	84.18
CTRL_1	50,367,716	45,816,685 (90.96%)	87.59	84.63
ET_0	52,994,877	49,072,336 (92.60%)	85.31	81.44
ET_1	49,632,631	45,108,436 (90.88%)	85.03	81.33
JA_0	48,741,461	44,148,196 (90.58%)	88.86	85.67
JA_1	50,592,096	45,832,497 (90.59%)	84.07	80.03
LA_0	49,293,920	44,946,197 (91.18%)	86.49	82.96
LA_1	46,807,234	42,014,823 (89.76%)	85.57	81.75

Reads were trimmed at quality threshold, Q20 with a minimum length of 100 bp using Cutadapt v1.7.1. Overall reads mapping and reads alignment was performed using Tophat v2.1.0.

### Gene expression profiling and differential gene expression analysis

Based on ssRNA-seq analysis, the pattern of fragments per kilobase of transcript per million mapped fragments (FPKM) distribution is almost similar across the samples where the majority of the genes fall between the ranges of 1 to 100. There are 27,815, 27,995, 29,565, and 29,366 genes in the CTRL, ET-, JA-, and LA-treated samples respectively that fall within this FPKM range (Supplementary Fig. S2a). The secondary laticifer containing from both JA- and LA-treated, had the most similar expression profile (Supplementary Fig. S3) compared with those without induced laticifer illustrating that the differences in the gene expression profile of JA- and LA-treated samples drive the phenotypic differences between samples. These findings are further supported by the similar expression patterns depicted in expression plots, such as the dendrogram, scatter matrix plot, volcano matrix plot, and correlation plot (Supplementary Fig. S2b–e).

**Figure 3:**
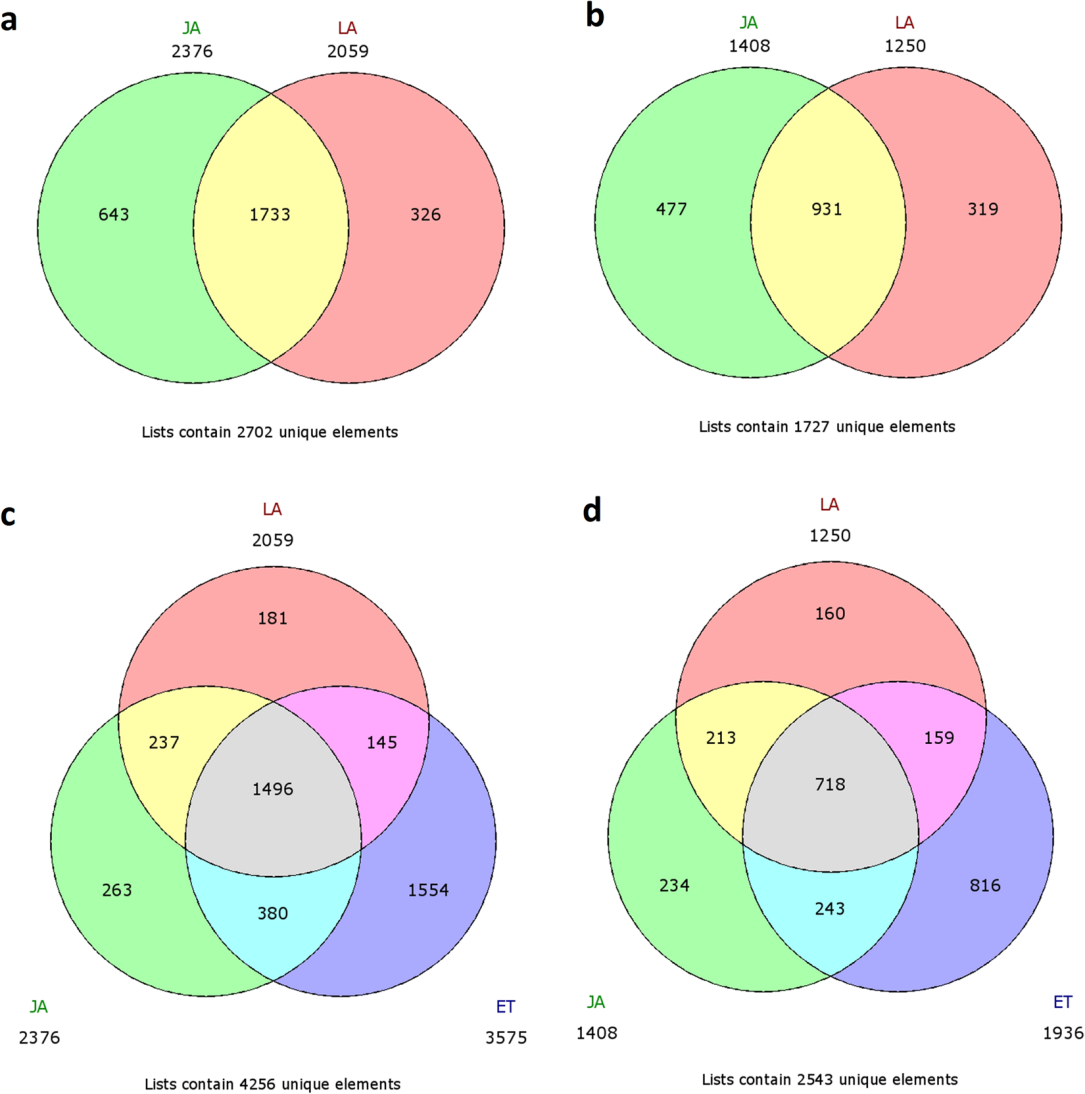
The Venn diagram of the DEGs (log_2_base 1 fold change) among the treated samples as compared to CTRL. The Venn diagram of upregulated genes (**a**) and downregulated genes (**b**) in both JA and LA samples as compared to CTRL. The Venn diagram of upregulated genes (**c**) and downregulated genes (**d**) in treated samples (JA, LA, and ET) as compared to CTRL. The yellow region in (**a**), (**b**), (**c**), (**d**) is assigned as JALAUP, JALADO, FUP, and FDO dataset respectively.

Compared with the CTRL samples, we identified 3,784, 3,309, and 5,511 differentially expressed genes (DEGs) in the JA-, LA-, and ET-treated samples respectively (Fig. 3). There were 2,664 DEGs shared in both the JA and LA dataset; herein referred as the JALA dataset (Fig. 3a,b). This could have been due to the similarly induced pathways by exogenous JA and LA^27^. Additionally, there were DEGs shared in the ET dataset with JALA dataset (Fig. 3c,d) indicating there is an interaction between JA and ET signalling as reported elsewhere^41,42^. Thus, filtering the JALA dataset with the ET dataset that could not induce SLD provided a catalogue of 450 DEGs (named as FDE dataset) for identifying the key regulatory genes directly associated with SLD.

### Putative novel transcripts detection in the JALA dataset

Compared with the annotated rubber reference genome, more than half of the transcripts in the JALA dataset (4,212 or 61.02%) had a partial match to the rubber draft genome. Of these, 717 transcripts were found in the FDE dataset (Table 2; Supplementary Table S4). These transcripts were coded ‘j’, ‘o’ and ‘u’ by using Cufflinks v2.2.1. ‘j’ indicates potentially novel isoform with at least one splice junction is shared with a reference transcript; ‘o’ indicates generic exonic overlap with a reference transcript whereas ‘u’ indicates unknown, intergenic transcript. Most of these putative novel transcripts shared different splice sites (90.38%), but a minority of them (5.15%) contained different transcribed exons of the same strand suggesting possible novel isoforms. Additionally, there were 188 unannotated transcripts (47 transcripts in FDE dataset) that were mapped between annotated gene regions of the rubber draft genome^34^, showing possible novel intergenic transcripts. Among these, 49 transcripts (14 transcripts in FDE dataset) had high sequence homology by BLAST (e ≤ 10^−5^) against the Swiss-Prot or the nr database (Supplementary Table S5) which further extends the current rubber tree gene prediction database^43^. The transcripts that had no match in any database appear to be unique to *H. brasiliensis* and we refer them as possible novel intergenic transcripts. The ssRNA-seq approach used in this study has provided useful to identify novel transcripts and functionally characterize genes that are associated to SLD as represented by the FDE dataset.

**Table 2:** The number of transcripts with strand information in class code assigned using Cufflinks v2.2.1 in each dataset.

Strand orientation	JALAUP		JALADO		FUP		FDO	
Class code	+	−	+	−	+	−	+	−
=	865	874	462	490	106	114	97	115
j	1,096	1,038	858	815	156	212	104	163
o	76	83	32	26	7	13	5	10
u	61	64	26	37	15	15	11	6
x	4	3	0	4	0	0	0	0

Class code ‘=’ indicates the complete match of the intron chain; ‘j’ indicates potentially novel isoform with at least one splice junction is shared with a reference transcript; ‘o’ indicates generic exonic overlap with a reference transcript; ‘u’ indicates unknown, intergenic transcript; ‘x’ indicates exonic overlap with reference on the opposite strand. Details are listed in Supplementary Table S4. ‘+’ symbolized sense transcripts whereas ‘−’ symbolized antisense transcripts. JALAUP and JALADO refer to the upregulated genes and downregulated genes in the JALA dataset respectively. FUP and FDO refer to the upregulated genes and downregulated genes in the FDE dataset, respectively.

### Discovery of AS events in Hevea

Many developmental processes and responses to stresses have been shown to be controlled by AS^44^. In this study, approximately one-fourth of the expressed genes are alternatively spliced in all samples (Table 3b). A total of 17,507 expressed genes display 94,945 AS events. The number of AS events is more than the number of expressed genes as some genes have multiple AS events. Applying the ASTALAVISTA tool to all samples indicates that intron retention is the most prevalent type among all of the quantified AS events, consistent with prior scientific reports^45,46^. It represents more than 38.51% of all AS events in each experimental condition, but this percentage is not as high as the 56% of the total events reported in *Arabidopsis* and *Oryza*^47^. We still expect that this percentage is underestimated because we used only bark material under four conditions and some AS events could be missing. The 3′ splice site was used 2-fold more frequently than the 5′ splice site was, in agreement with other studies^45,47^. In addition, exon skipping is a rare event in the assembled transcripts (Table 3; Supplementary Table S6). However, most of the FDE transcripts were spliced at the 3′ site, followed by multiple AS events and intron retention, indicating that altered AS events occurred after ET sample filtering and suggesting a direct role of specific splicing isoforms in SLD. These AS events of the randomly chosen TFs are depicted in the sashimi plots using the IGV tool and further support the ASTALAVISTA results (Table 4 Supplementary Fig. S4). Similarly, incidence of altered AS events have also been reported elsewhere such as in *Arabidopsis thaliana*^48,49^. Altered splicing events, which may lead to the expression of SLD-specific variants, are most likely promoted by alterations in the activity of *trans*-acting splicing regulators. Based on Eukaryotic Orthologous Group (KOG) analysis, splicing factor genes (U2 small nuclear RNA auxiliary factor 2, splicing factor 3B subunit 1, splicing factor 1) and the heterogeneous nuclear ribonucleoprotein F gene were detected in the FDE dataset, thus strengthening the idea that a change in the level of splicing regulators in induced secondary laticifer samples may impinge on gene expression networks. Functional studies have to be done on these variants to validate whether these changes in splicing profiles are artifacts occurring during the SLD or whether they play a direct role in SLD.

**Table 3:** The proportion of AS events and alternatively spliced transcripts identified using AStalavista 2.0 in each sample.

Sample AS type (%)	CTRL	ET	JA	LA	*FUP	*FDO	*merged.gtf
**(a)**							
Intron retention	40.62	38.51	42.65	42.05	23.14	23.97	20.32
Alternative 3′ acceptor site	28.41	31.13	26.03	26.30	30.18	30.60	26.28
Alternative 5′ donor site	12.57	13.23	11.79	12.09	16.90	15.46	14.85
Exon skipping	4.70	5.24	5.15	5.27	6.04	4.73	6.85
Other AS events	13.70	11.89	14.39	14.29	23.74	25.24	31.70
**(b)**							
Alternatively spliced transcripts (%)	34.46	33.20	35.61	36.15	45.77	78.67	41.23
Alternatively spliced unigenes (%)	28.04	26.21	28.13	30.21	49.79	42.72	18.82

(a) The proportion of certain AS type relative to the total number of AS events in each sample (b) The frequency of the spliced variants relative to the total expressed transcripts. Details for the dataset assigned with an asterisk are found in Supplementary Table S6. FUP and FDO refer to the upregulated genes and downregulated genes in the FDE dataset, respectively.

**Table 4:** The identified AS events of the selected TFs (sense transcripts) from the FDE dataset. The transcriptional events were visualized via IGV v2.3.80. The generated sashimi plots can be found in Supplementary Fig. S4.

FUP	Gene Name	AS type	Number of transcripts involved in AS events
1	MYB44	Alt acceptor	2
2	RAV1	Nil	0
3	NFYA3-like	Alt acceptor	2
4	MYB86	Alt acceptor	2
5	LBD1	Nil	0
6	AP2-like	Alt donor	2
7	bHLH13-like	Multiple AS events	3
8	SBP6	Alt acceptor, intron retention and other AS events	3
9	Trihelix	exon skipping	2
**FDO**	**Gene Name**	**AS**	**Number of transcripts involved in AS events**
10	ICE1	Nil	0
11	MYB1R1	intron retention	3
12	COL	Nil	0

### Gene prediction and annotation of the JALA dataset

Gene prediction and functional annotation of the JALA transcript list were performed against various databases such as the NCBI nucleotide collection, Swiss-Prot database, GO database, and InterPro database (Supplementary Tables S7 and S8). According to KOG analysis^50^, a total of 1,614 transcripts (76.13%) had specific KOG categories assigned, with categories such as ‘signal transduction mechanism’, ‘posttranslational modification, protein turnover, chaperone’, and ‘transcription’, which were overrepresented compared to the other assigned categories. The KOG analysis of relative abundance values suggested particularly intense signalling, transcriptional, and translational activities associated with the JA- and LA-induced SLD (Table 5, Supplementary Table S9). Here, we identified more than 10 DEGs in Ca^2+^ signal transduction and 8 DEGs in mitogen-activated protein kinase (MAPK) cascades. Ca^2+^ and MAPK genes are the key contributors to signal transduction mechanisms that regulate important cellular processes in plants^51,52^. Apart from Ca^2+^ and MAPK signal transduction, genes participating in the CLAVATA (CLV)-WUSCHEL-related homeobox (WOX) signalling pathway are differentially regulated in our study, as reported elsewhere^38^. The CLV signalling pathway is known to play a key role in stem cell homeostasis^53,54^ and is involved in plant immune responses^55,56^. CLV1 encodes a transmembrane receptor kinase and functions by binding to the (CLV3/Endosperm surrounding region-related) CLE peptide. This CLV3/CLE-CLV1 ligand-receptor kinase pair and WOX are known to act antagonistically^38,57^. Nonetheless, in this study, we found that the two unigenes encoding WOX (a key regulator of stem cell fate in different meristems)^58^ together with two unigenes encoding CLV1 were upregulated in all treated samples except for one CLV1-related gene is downregulated in the FDE dataset, thus implicating the complexity of the underlying regulatory mechanism. Taken together, the findings of the present study indicated a cross-link among JA and LA signalling, Ca^2+^ and MAPK signal transduction and CLV-WOX signalling pathway associated with SLD in the *Hevea* clone RRIM 600.

**Table 5:** KOG analysis of the JALA dataset with transcript counts and proportion relative to total transcripts in each assigned article. Details can be found in Supplementary Table S9.

KOG categories	Transcripts count	Percentage (%)
**Information storage and processing**		
[J] Translation, ribosomal structure and biogenesis	62	2.92
[A] RNA processing and modification	79	3.73
[K] Transcription	146	6.89
[L] Replication, recombination and repair	63	2.97
[B] Chromatin structure and dynamics	26	1.23
Total	376	17.74
**Cellular processes and signaling**		
[D] Cell cycle control, cell division, chromosome partitioning	65	3.07
[Y] Nuclear structure	16	0.75
[V] Defence mechanisms	18	0.85
[T] Signal transduction mechanisms	291	13.73
[M] Cell wall/membrane/envelope biogenesis	44	2.08
[N] Cell motility	3	0.14
[Z] Cytoskeleton	72	3.40
[W] Extracellular structures	23	1.08
[U] Intracellular trafficking, secretion, and vesicular transport	89	4.20
[O] Posttranslational modification, protein turnover, chaperones	150	7.08
Total	771	36.37
**Metabolism**		
[C] Energy production and conversion	40	1.89
[G] Carbohydrate transport and metabolism	118	5.57
[E] Amino acid transport and metabolism	58	2.74
[F] Nucleotide transport and metabolism	20	0.94
[H] Coenzyme transport and metabolism	16	0.75
[I] Lipid transport and metabolism	90	4.25
[P] Inorganic ion transport and metabolism	90	4.25
[Q] Secondary metabolites biosynthesis, transport, and catabolism	35	1.65
Total	467	22.03
**Poorly characterised**		
[R] General function prediction only	288	13.58
[S] Function unknown	218	10.28
Total	506	23.87

### Identification and classification of TFs and transcriptional regulators (TRs) using iTAK

A few TF gene families have been reported to be important in the SLD. A few members of the AP2/ERF superfamily, *HbERF1*, *HbERF2*, *HbERF3*, and *HbRAV1* genes, were differentially expressed during JA-induced laticifer differentiation in the *H. brasiliensis* clone 7-33-97^59^. Other reported TF gene families associated with SLD in *H. brasiliensis* clone RRIM 2016 were ring finger, zinc finger, and MYB^32,60^. Of the 89 identified TF and TR subfamilies in the rubber draft genome^34^, 26 subfamilies were identified in the FDE dataset using the iTAK pipeline^61^. The most abundant TF subfamilies in the FDE dataset are MYB (7 genes), AP2/ERF (5 genes), basic-helix-loop-helix (bHLH) (3 genes), zinc finger (3 genes), GRAS (2 genes), plant AT-rich sequence- and zinc-binding protein (PLATZ) (2 genes), and SQUAMOSA Promoter-Binding Protein-Like (SBP) (2 genes) (Supplementary Table S10), suggesting the potential involvement of these TFs in *Hevea* bark SLD. Our results are similar to those of a previous study, in which bHLH TFs were found to be the master regulators of JA-responsive gene, whereas ERF and MYB TFs fine-tune the JA-mediated gene expression in *Arabidopsis thaliana*^62^.

Upon the degradation of Jasmonate Zim (zinc-finger inflorescence meristem) domain (JAZ) gene by the ubiquitin/26S proteasome-dependent proteolytic pathway, a bHLH type TF, MYC2 acts together with MYC3 and MYC4 to activate JA-responsive genes by directly targeting their promoters^63–65^. MYC2, one of the most intensively studied MYC members that acts upstream in the JA signalling pathway^66^, is differentially expressed in all treated samples compared with the CTRL in the current study. In addition, other MYC homologous unigenes were annotated as MYC1, MYC3, MYC4, and MYC anthocyanin regulatory proteins in the JALA dataset. All these MYC homologues were also differentially regulated by exogenous ET, demonstrating their involvement in the ET signalling pathway in rubber trees as well. This is supported by other scientific evidence showing that MYC genes are involved in ethylene signalling in *Arabidopsis*^67^. Although the current evidence shows that these MYCs act as master regulators on the onset of JA-responsive gene expression, additional work must be done to reveal the dense downstream of JA-signalling circuitry and its role in SLD. MYC2-like 2 that acts as a repressor protein in JA-mediated plant defence and development^68^, is upregulated in the FDE dataset, which may be due to the feedback mechanism induced by the jasmonate response. In terms of tissue differentiation, another bHLH-type TF, the ICE1 gene, has been reported to interact with the ICE2 gene, leading to stomatal differentiation in *Arabidopsis*^69^. Similarly, the DAG protein is known to be involved in plant cell differentiation^70^. In the present study, we found that both ICE1 and DAG gene were downregulated in the FDE dataset, which suggests their role in the differentiation of the secondary laticifer.

Previous studies reported that JAZ genes are differentially regulated by jasmonates and coronatine^38,71–74^. Under normal conditions, JAZ repressor proteins interact and inhibit the action of TFs that regulate JA-mediated genes^26,72^. During JA-mediated signalling, JAZ is degraded by ubiquitin-mediated proteolysis resulting the release of JAZ-bound TFs and subsequent transcriptional activation^73,75–77^. Although several JAZ homologues were isolated from the latex of rubber trees^78–80^, little is known about their roles in SLD. In the present study, some of these JAZ homologues were annotated as JAZ2, JAZ7 till JAZ11, but only JAZ 2, JAZ 8, and plastid jasmonates ZIM-domain protein were upregulated in the JALA dataset. These JAZ homologues are also differentially regulated by ET, which indicates that they are not specific to JA and LA responses. Although JAZ genes have been identified as the core component of the JA signalling pathway, only a few of them were differentially expressed in the JALA dataset. This could have been due to the effects of the JA and LA treatment on gene expression being missed at early harvesting time points.

Besides MYC and JAZ proteins, the three amino acid loop extension (TALE) superclass homeobox genes are also associated to plant tissue development. KNOTTED-like homeobox (KNOX) and Bel1-like proteins under this TALE homeodomain superclass were reported forming heterodimers in barley^81^, *Arabidopsis*^82,83^, and maize^84^. The KNOX family of TFs plays a key role in controlling the shoot apical meristem activity such as boundary establishment, the correct patterning of organ initiation, the development of axillary meristems, and contribute to the leaf form diversity^85^ whereas Bel1 involved in tuber formation in potato^86^. In this study, two KNOX genes are upregulated in the JALA dataset, and there is one Bel1 homolog gene downregulated in the FDE dataset. In accordance with another study, KNOX TFs were reported to be regulated by ethylene^87^. These genes were also suggested to be involved in the gibberellic acid (GA) signalling pathway^88^. Here, we found more than 10 genes in the JALA dataset (GRAS TFs, gibberellic acid 2-oxidase, chitin-inducible gibberellin-responsive 1-like, gibberellin receptor GID1B-like, gibberellin-regulated 3 precursor, and geranyl diphosphate synthase, SHI related sequence and TALE homeobox genes) that are related to GA signalling cascades, suggesting that overlapping components are shared among the JA, LA, and GA signalling pathways. In addition, the regulators of the KNOX gene in our study were in accordance with other studies: Positive regulators of KNOX genes from the CUP-SHAPED COTYLEDON (CUC) gene family, encoding the NAC (NAM ATAF1 CUC2) TFs^89–92^ were upregulated in the JALA dataset, whereas the negative regulators such as BLADE ON PETIOLE (BOB1 and BOB2 containing BTB/POZ domain)^93^ and axial regulator YABBY genes^94^ are downregulated in the FDE dataset. Previous report has documented that the Bel1-like or Bel5, GA2 oxidase 1 (GA2ox1), and KNOX genes regulate tuber formation in potato^86,88^. Based on these findings, it is possible that Bel5-GA2ox1-KNOX may act together in the secondary laticifer development in *Hevea*.

On the other hand, TRs regulate the expression of target genes either via indirect interaction with the TFs or by altering the accessibility of DNA to TFs via chromatin remodelling. Therefore, identification and classification of these TRs is important for understanding the regulatory networks in SLD. Six groups of TRs, Pseudo phospho-accepting response regulator (ARR), Tumor necrosis factor receptor-associated factor (TRAF), Tafazzin (TAZ), Gcn5-related N-acetyltransferase (GNAT), Orphan genes, and Sucrose non-fermenting (SNF2) were identified by using the iTAK program (Supplementary Table S10). Two Pseudo ARRs, namely APRR3 and APRR5, are found downregulated in the FDE dataset. The Pseudo ARRs are known to be involved in the phosphorelay signal transduction, regulation of transcription, regulation of flowering time, photomorphogenesis, and rhythmic process of *Arabidopsis thaliana*^95–97^. In addition to this, the Gigantea gene and zinc finger CONSTANS TF involved in the plant circadian rhythmic process^98^ are found differentially regulated in the same dataset. This result is in line with recent finding demonstrating that the basal levels of jasmonates in *Arabidopsis* are under the control of the circadian clock for enhanced resistance against a generalist herbivore^99^. In addition to this, another study has reported that Arachidonic acid-induced DEA1 gene that involved in the circadian regulation can be induced by external chemical treatment and pathogen attack in *Solanum*^100^. Thus, the application of JA and LA treatment might trigger changes in the basal levels of jasmonate in the *Hevea* plant, which in turn affects the expression of these circadian rhythm-regulated genes. Four CCT domain-containing genes, CONSTANS-LIKE (COL), Pseudo ARR, GATA-type zinc finger, and Orphans, were differentially expressed in FDE dataset. CCT domain containing genes generally control flowering in plants^101,102^. Another gene, DNA-binding protein phosphatase 1 (*AtDBP1*), that acts a promoter of flowering^103^ is found downregulated in the FDE dataset. This result implies that exogenous JA could affect the expression of flowering genes in *Hevea*. In addition to its role in flowering, DBP1 mediates susceptibility to two potyviruses in *Arabidopsis*^104–106^. Besides directly participating in transcriptional regulation of specific genes by virtue of its DNA-binding capacity, the dual structure of DBP factors suggests that they may also be involved in the regulation of other processes such as signal transduction pathways^104^. This hypothesis was supported by the dynamic localization of the tobacco *NtDBP1* protein for which a shuttling mechanism from the nucleus to the cytosol has recently been shown^107^. Further analysis needs to be done on its dual structure to unravel its role in SLD.

Another TR identified is TRAF was found downregulated in the FDE dataset. Two *Arabidopsis* TRAF domain-containing proteins (MUSE13 and MUSE14) function together with E3 ubiquitin ligase SCFCPR1 in regulating the turnover of leucine-rich repeat-containing (NLR) immune receptors^108^, but in mammals, it has been shown that these proteins play indispensable roles in innate and adaptive immunity, development, and abiotic stress responses^109^. Our results are in accordance with those of other studies where the JA level was inversely proportional to the expression of TRAF^110,111^; however, we are the first to report its possible involvement in SLD. Besides this, we found three genes (TAZ, GNAT, and SNF2) in the FDE dataset that are related to histone acetylation. The specific patterns of histone acetylation could alter the rate of chromatin remodelling in many ways^112^ and in particular, a histone deacetylase inhibitor, Trichostatin A, was reported to induce induce SLD in *H. brasiliensis*^24^. This further suggests the importance of histone acetylation in the regulation of SLD.

### The relative expression of selected TFs in the JA-treated *Hevea* clones

**Figure 4:**
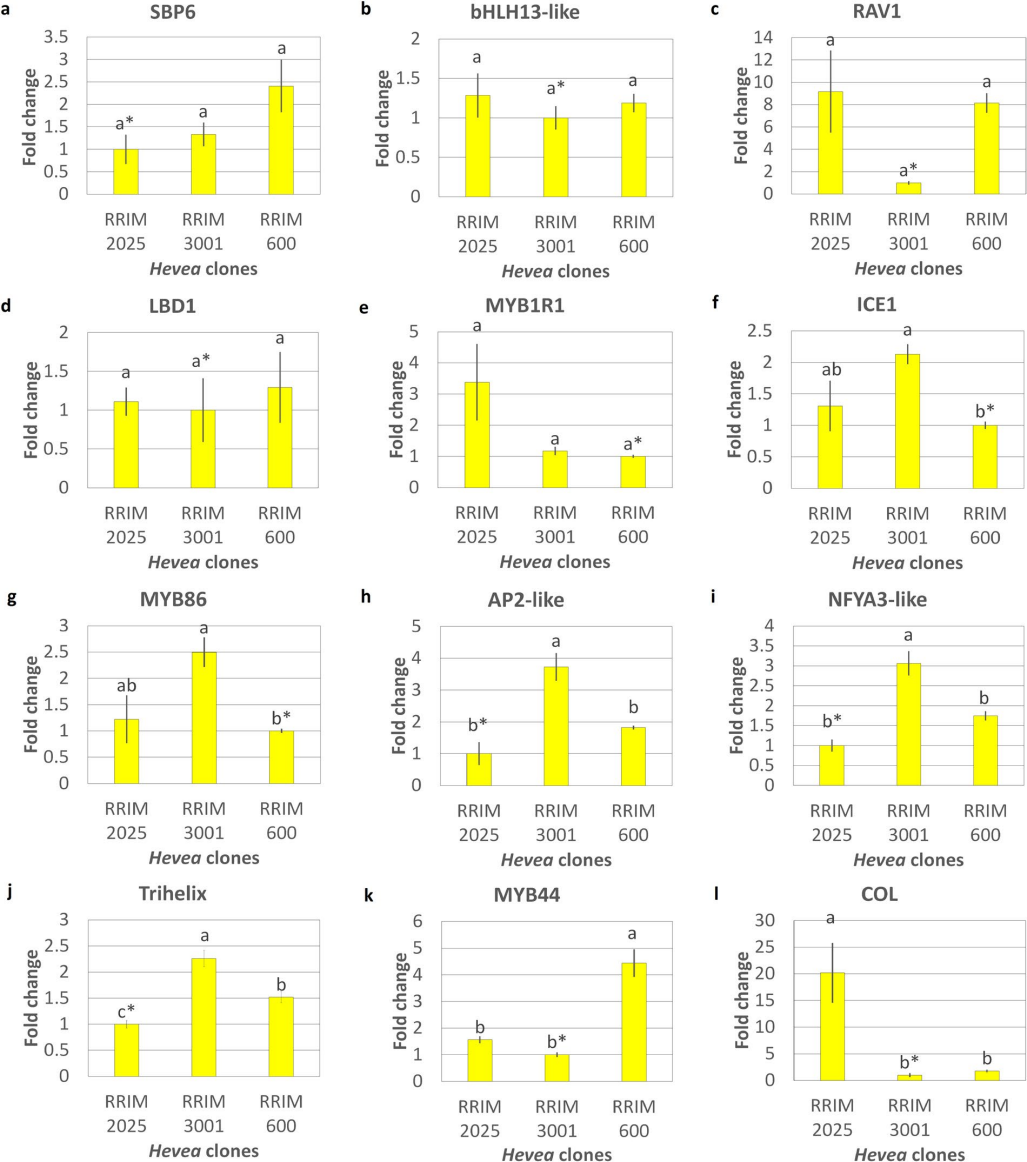
The relative fold change expression of each TF among JA-treated *Hevea* clones by using relative qRT-PCR analysis. Reference sample is indicated by asterisk in each image and ADF4 is used as reference gene. The significance in relative expression of TFs was tested through a one-way ANOVA, followed by Tukey’s post hoc test at a significance level of 0.05. Each vertical line on the bar represents mean ± SEM across the biological replicates. Mean values with different letters are significantly different.

Understanding the expression patterns of TFs is of particular interest because the activity of these regulatory proteins is crucial for controlling the expression of numerous genes and, thus, for controlling and coordinating these diverse pathways and processes. By using relative qRT-PCR, TFs such as AP2-like, ICE1, trihelix, MYB86, and Nuclear transcription factor Y subunit A3-like (NFYA3-like) had similar relative gene expression patterns (Fig. 4), in correlation with the induced secondary laticifer line number among the JA-treated clones (Fig. 2a), implicating their possible roles in regulating the size of the induced secondary laticifer line number. The results from the qRT-PCR experiment also validated the ssRNA-Seq expression data and therefore corroborated the robustness of the findings. Consequently, these five TFs are suggested to play an important role in *Hevea* secondary laticifer development. Of the 12 tested TFs, 5 ((MYB1R1, SBP6, bHLH13-like, Related to ABI3/VP1 (RAV1), and LBD1)) were not differentially expressed in all of the clones, suggesting that the expression of these TFs has a similar magnitude towards JA-induced responses across all tested clones. Interestingly, the relative gene expression of the COL gene was at least 11-fold higher in the JA-treated RRIM 2025 than it was in other clones, which indicates that the high expression of this TF in JA-treated RRIM 2025 might be clone-dependent. This CCT domain containing gene generally control flowering^101,102^ and floral meristem identity in plants^113^. Flower is one of the plant components that is used for plant classification and recognition^114^. Thus, exogenous JA could possibly affect the flowering pathway to different extent that it may give rise to unique variations among the tested clones.

### Data comparison and cross-validation of ssRNA-seq data

For cross-data validation, we compared the relative gene expression profiles of JA-treated samples from two different platforms: ssRNA-seq and relative qRT-PCR from our current study (Supplementary Fig. S5). Gene expression for selected genes was normalized relative to trihelix gene. For JA-treated samples, with exception to NFYA3-like, the fold change expression patterns of all other selected genes were similar between the two data sets (91.67%). In addition, we also provide comparative data between ssRNA-seq data from the current study and our previous work using microarray data for all experimental conditions (i.e. control, JA, LA, and ET) (Loh *et al*. 2016). We randomly selected 14 FDE genes identified in both studies for validation purpose (Supplementary Fig. S6). 12 upregulated genes in the FDE dataset (FUP) were validated because they showed similar fold change patterns to those in the ssRNA-seq data where they were higher in the JA- and LA-treated samples compared with other conditions. However, the expression of the genes encoding basic-leucine zipper (bZIP) and TRAF, showed different fold change patterns. In our previous microarray analysis^32^, bZIP and TRAF were upregulated in both the JA- and LA-treated groups compared with the other groups; however, they were downregulated in the ssRNA-seq analysis. 85.71% of the selected gene expression patterns were verified by comparing with our previous microarray dataset.

## Conclusion

The findings of our study provided a fundamental framework on the identification and molecular characterization of transcriptional activities directly related to SLD in *H. brasiliensis*. Our results showed a potential relationship between the JA, LA, ET, Ca^2+^, CLV-MAPK-WOX, and GA-TALE signalling pathway in the regulation of JA- and LA-induced SLD in the rubber tree RRIM 600. Most of the TFs in the FDE dataset are classified under bHLH, MYB, and AP2/ERF gene families, thus suggesting the importance of these gene families in the regulation of SLD. Future works should be targeted at characterizing the functions of the identified regulators in the FDE dataset that were previously not linked to SLD.

## Material and Methods

### Plant material and treatments

Young *H. brasiliensis* RRIM 600 plants with 3 extension units (3 layers stage) were grown under natural light in the experiment garden of the Centre for Chemical Biology, Universiti Sains Malaysia. Fifteen plants were treated with JA, LA, and ET respectively for the top 2 extension units^18^. The stem was scarified with a sharp razor on the stem surface approximately 1 cm^2^ directly below the lowest foliage of the extension unit. 3% of chemical-premix in lanolin paste was applied on the wounded region and was covered with polyethylene membrane. The 3% of chemical-premix in lanolin paste was expressed as the weight percentage in the lanolin paste containing the chemicals. Fifteen untreated plants of the same age were grown as controls. A more detailed description of the plant treatment procedure can be found in our previous works^32^. For ssRNA-seq data validation, 30 *Hevea* plants per clone (RRIM 2025, RRIM 3001, and RRIM 600) were treated with 3% JA-premix in lanolin paste. For all experiments, samples were harvested after 2 months of the treatments^18^. Sample collection procedures were done according to our previous work^32^.

### RNA library preparation and sequencing

The total RNA samples from the bark region were prepared according to our previous work^32^ and outsourced to Macrogen Inc., Korea, for sequencing with Illumina TruSeq Stranded mRNA sample Prep kits after poly-A tailing enrichment. The prepared cDNA library size and concentration were determined using an Agilent Technology 2100 Bioanalyzer with D1000 screen tape. The library size ranged from 271 to 293 bp. RNA extracted from 8 plants (bark region) were pooled for each biological replicate. Two independent biological replicates were used for each experimental conditions (i.e. control, JA, LA, and ET) resulting in a total of eight prepared libraries. Four libraries each were multiplexed in a single lane of the Illumina sequencing run. 30% of the PhiX controls were added per sample to avoid bias of the GC content. The libraries were then sequenced using an Illumina Hiseq2500 sequencing system in rapid-run mode, which generated 150 bp paired-end reads. We targeted a high sequencing depth approach on the ssRNA-seq instead of targeting a higher number of biological replicates as greater sequencing depth would provide maximum power to detect more informational reads^115^, including those with weak expressions, for the differential expression analysis. In addition, estimation accuracy for low expression genes is known to be improved by adding sequencing depth^116^.

### Data preprocessing and mapping

FastQC v0.11.2 was used to check the quality of the sequenced reads before and after trimming. Cutadapt v1.7.1 was utilized to trim the adaptor, reads below 100 bp and reads with PHRED quality scores less than 20^117,118^. High quality trimmed reads were then used for downstream data analysis.

### Data analysis

The ssRNA-seq data analysis was performed using Tuxedo pipelines in the open-source software packages Bowtie v2.2.6, Tophat v2.1.0, and Cufflinks v2.2.1^119,120^. The transcript assembly was accomplished by incorporating the recently published rubber draft genome as a reference^34^. All BAM files were used as input for Cuffmerge, which assembles parsimonious consensus fragments from the BAM file coordinates^121^. With the use of ASTALAVISTA tool v2, AS events were identified^122^. The Cuffdiff module of the Cufflinks software takes the aligned reads in (FPKM) of two or more conditions reporting DEGs and isoforms. It adopts an algorithm that controls cross-replicate variability and read-mapping ambiguity by using a model for fragment counts based on a beta negative binomial distribution^123^. The reads were normalized to FPKM using a fragment normalization constant at 36,432,000 with the internal scale shown in Supplementary Table S11 for quantification analysis. The DEG was selected in each treated sample compared with CTRL using the following criteria: FPKM > 0, p value < 0.05, FDR < 0.05, and fold change ≥ 2^124^ and presented using online Venn diagram software (http://www.bioinformatics.lu/venn.php). The DEGs common in both JA- and LA-treated bark samples compared with CTRL were selected as the JALA dataset. To sort the FDE dataset, we filtered the JALA dataset with DEGs between ET-treated bark samples and CTRL. These two datasets were used for downstream data analysis. Gene prediction and functional annotations of the JALA dataset were accomplished based on the NCBI nucleotide collection^125^, Swiss-Prot^126^, GO^127,128^, InterPro^129^, and KOG^130^ databases. A minimum of E value cutoff at 1e-5 was applied to the searches against all databases. Gene prediction was performed through BLASTX searches against the NCBI non-redundant database via Blast2GO Basic v3.0 (database Feb 2016)^131^. The gene prediction was done by combining the results from the Swiss-Prot and NCBI non-redundant database, for which the data from the Swiss-Prot database were chosen as the main gene prediction data. GO analysis was applied in Blast2GO to analyse the main function of DEGs in the JALA dataset^128,132^. These protein sequences were further functionally annotated for conserved domain analysis against the KOG database^133^. In addition, the InterPro annotations in Blast2GO were used as the domain information^134^. The TFs and TRs in the JALA dataset were identified and classified into families using the iTAK pipeline (http://bioinfo.bti.cornell.edu/tool/itak)^61^.

### Data visualization and integration

CummeRbund v2.12.1 was installed in an R statistical computing environment (R Bioconductor v3.2.3) to generate expression plots such as a density plot, scatter plot, volcano matrix plot, and heatmaps, and to visualize the distribution pattern of the expressed genes across all experimental conditions. The Jensen-Shannon (JS) distance was used to create the dendrogram and heatmaps. The gene expression values in the heatmap were presented in shaded colours where darker colours indicate higher expression values. Sample trees were drawn horizontally, whereas transcript trees were drawn vertically. In addition, a Pearson’s correlation test was performed using Corrplot v0.73 to measure the correlation between sample pairs^119^. Transcriptional events of the selected TFs across the samples against the reference genome were visualized using the IGV tool v2.3.80^135^.

### Relative qRT-PCR analysis

The relative qRT-PCR experiment was conducted to observe the relative expression of the selected TFs in relation to the different types of *Hevea* clones in order to see if the results observed in RRIM600 are also reproducible in other *Hevea* clones and to explore if there is any TFs that potentially expressed differently across different clones therefore potentially influence the phenotypic characteristics of the clones associated to secondary laticifer development (i.e. high latex yielding clone versus low latex yielding clones). Nine upregulated and three downregulated unigenes in the FDE dataset were chosen for relative qRT-PCR analysis across different *Hevea* clones. The JA-treated *Hevea* clones were RRIM 600, RRIM 2025, and RRIM 3001. Since both JA and LA can induce secondary laticifer, we chose only JA treated plants as a representative to evaluate the expression patterns of the selected TFs across the different *Hevea* clones. The relative gene expression level of the TFs among these *Hevea* clones was then used to correlate with the DRC and JA-induced secondary laticifer line number. Relative quantification was performed using samples from three independent replicates. For each biological replicate, three technical replicates were performed. Each cycle threshold (Ct) mean was calculated by using these three Ct values. Samples with the lowest gene expression level were used as the reference. A Ct of 10,000 was used for all experimental run. Relative expression of the genes was measured using the 2^−ΔΔCt^ method^136^. A more detailed procedure for designing primers, generating standard curves, and performing 2-step relative qRT-PCR can be found in our previous work^32^. The PCR efficiencies (E) for each standard curve were between 96% and 105% with R^2^ values higher than 0.95. The Actin depolymerizing factor 4 (ADF4) gene is one of the top five housekeeping genes suggested for *Hevea* samples^137^. Moreover, its expression was shown to be constant across all experimental conditions in the ssRNA-seq analysis. Thus, ADF4 was used in this study as an internal control and amplified in parallel with the target genes. All primer sequences are presented in Supplementary Table S12. By using the SPSS v16.0 software, the significance in relative expression of TFs was tested through a one-way analysis of variance (ANOVA), followed by Tukey’s post hoc test at a significance level of 0.05.

### Determination of DRC

To correlate the relative expression of selected TFs with the DRC, we implemented ISO 126:2008^138^ method to determine the DRC of natural rubber latex concentrate in the JA-treated RRIM 2025, RRIM 3001, and RRIM 600 bark samples. The DRC of each clone was carried out in 3 biological replicates. First, 100 µL of fresh latex was collected from the JA-treated region per bark sample into a pre-weighed tube. Each latex sample was preserved in 10 µL of ammonia (1 M) and diluted with distilled water to a 20% total solid content (ISO 124:2011). Then, 350 µL of 20 g/dm^3^ acetic acid aqueous solution was added to the diluted latex sample for latex coagulation. The coagulum was pressed by a hand homogenizer, forming a sheet less than 1 mm thick. If the solution remained milky, 50 µL of 95% EtOH was added. All of the supernatant was removed before rinsing with distilled water until the coagulum was not acidic. The coagulum was then dried at approximately 70 °C in an oven until there were no white patches. It was allowed to cool in a desiccator before weighing. The procedure of drying, cooling, and weighing was repeated until the mass was constant. The DRC of the latex was given by the percentage of weight of the dry sheet over the weight of the fresh latex. Comparisons between the DRCs of various samples were performed out through a one-way ANOVA, followed by Tukey’s post-hoc test at a significance level of 0.05.

### The quantification of the secondary laticifer line number and primary laticifer distribution

Tissue collection and histochemical staining methods to determine the laticifer line numbers were done according to our previous work^32^. Latex and lipid-soluble substances were stained with 50 mg/mL Nile red (Sigma-Aldrich Corp., MO, US) in 50% ethanol for approximately 10 s. The sections were mounted in VECTASHIELD mounting medium (Vector Laboratories Inc., CA, US) and then sealed with nail varnish^8,139^. The secondary laticifer line number and primary laticifer distribution of the JA-treated *Hevea* bark samples (RRIM 2025, RRIM 3001, and RRIM 600) were measured under fluorescence-microscopic observation. Within the cryosectioned bark samples, the secondary laticifer running along the entire width was expressed as 1 secondary laticifer line, but a value less than 1 indicate that the secondary laticifer did not run along the entire width^18^. The primary laticifer distribution indicates the mean area of primary laticifers in the primary tissue in a 1 mm^2^ cross-section of bark sample. Data were collected from 3 randomly chosen locations in a section for each sample and 3 biological replicates per clone^18,140^. A constant area of 0.48 mm^2^ was viewed on each image to determine laticifer distribution. All measurements were performed using the Olympus Cell^F imaging software. Comparisons between the secondary laticifer line number and primary laticifer distribution of various samples were performed through a one-way ANOVA, followed by Tukey’s post-hoc test at a significance level of 0.05.

## Supplementary information

All Supplementary Materials except Table S3,4,6-9

Supplementary Table 3

Supplementary Table 4

Supplementary Table 6

Supplementary Table 7

Supplementary Table 8

Supplementary Table 9

## Data Availability

The raw sequencing data was deposited at: https://www.ncbi.nlm.nih.gov/sra/SRP157878 under accession SRP157878. The supplementary information from the current study is available at: https://drive.google.com/open?id=1GqLsZubqflGFS8nBPRJxpuI_hnlLDozQ.
