# Supplementary material for: Identification and characterization of jasmonic acid- and linolenic acid-mediated transcriptional regulation of secondary laticifer differentiation in Hevea brasiliensis
Source: Sci Rep. 2019 Oct 4;9:14296. doi: 10.1038/s41598-019-50800-1 (PMC6778104; doi:10.1038/s41598-019-50800-1)
Supplement: Supplementary file 1 — All Supplementary Materials except Table S3,4,6-9 [file 41598_2019_50800_MOESM1_ESM.pdf]

Identification and characterization of jasmonic acid- and linolenic acid-mediated transcriptional regulation of secondary laticifer differentiation in *Hevea brasiliensis*

Shorter running title: Molecular basis of secondary laticifer differentiation in *Hevea*

Swee Cheng Loh[1], Ahmad Sofiman Othman[1, 2], G. Veera Singham[1*]

[1] Centre for Chemical Biology, Universiti Sains Malaysia, 11900 Bayan Lepas, Penang, Malaysia.

[2] School of Biological Sciences, Universiti Sains Malaysia, 11800 Penang, Malaysia.

Email (first author): lohsweecheng87@gmail.com

Email (second author): sofiman@usm.my, contact number: (+6) 04 653 4019

Email (corresponding author): veerasingham@usm.my, contact number: (+6) 04 653 5534

*Corresponding author

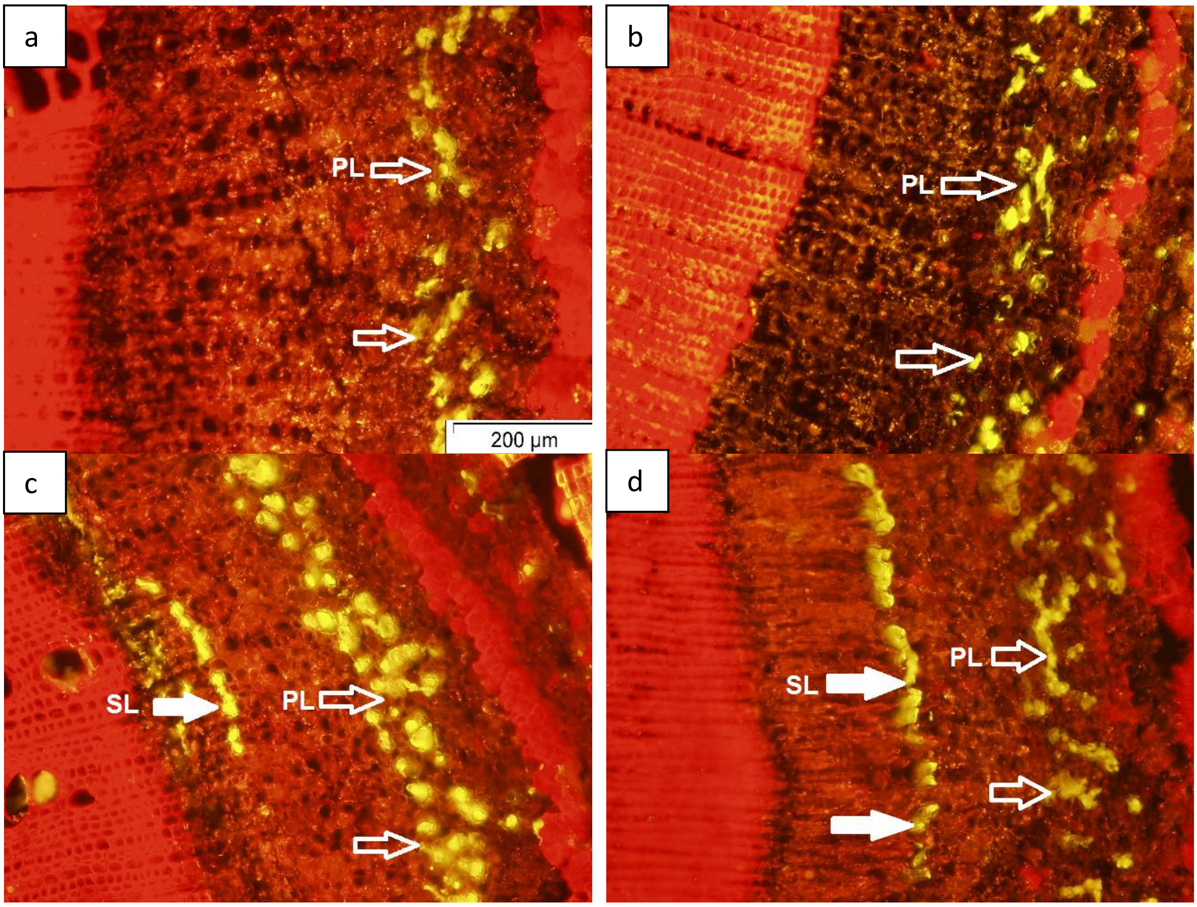

Supplementary Figure 1. Comparison of transverse sections of *H. brasiliensis* bark samples, clone RRIM 600, under different experimental conditions: (a) CTRL (b) ET-treated (c) JA-treated (d) LA-treated samples. White outlined arrows indicate primary laticifer (PL) while white arrows indicate induced secondary laticifer (SL). Scale bar: 200 µm.

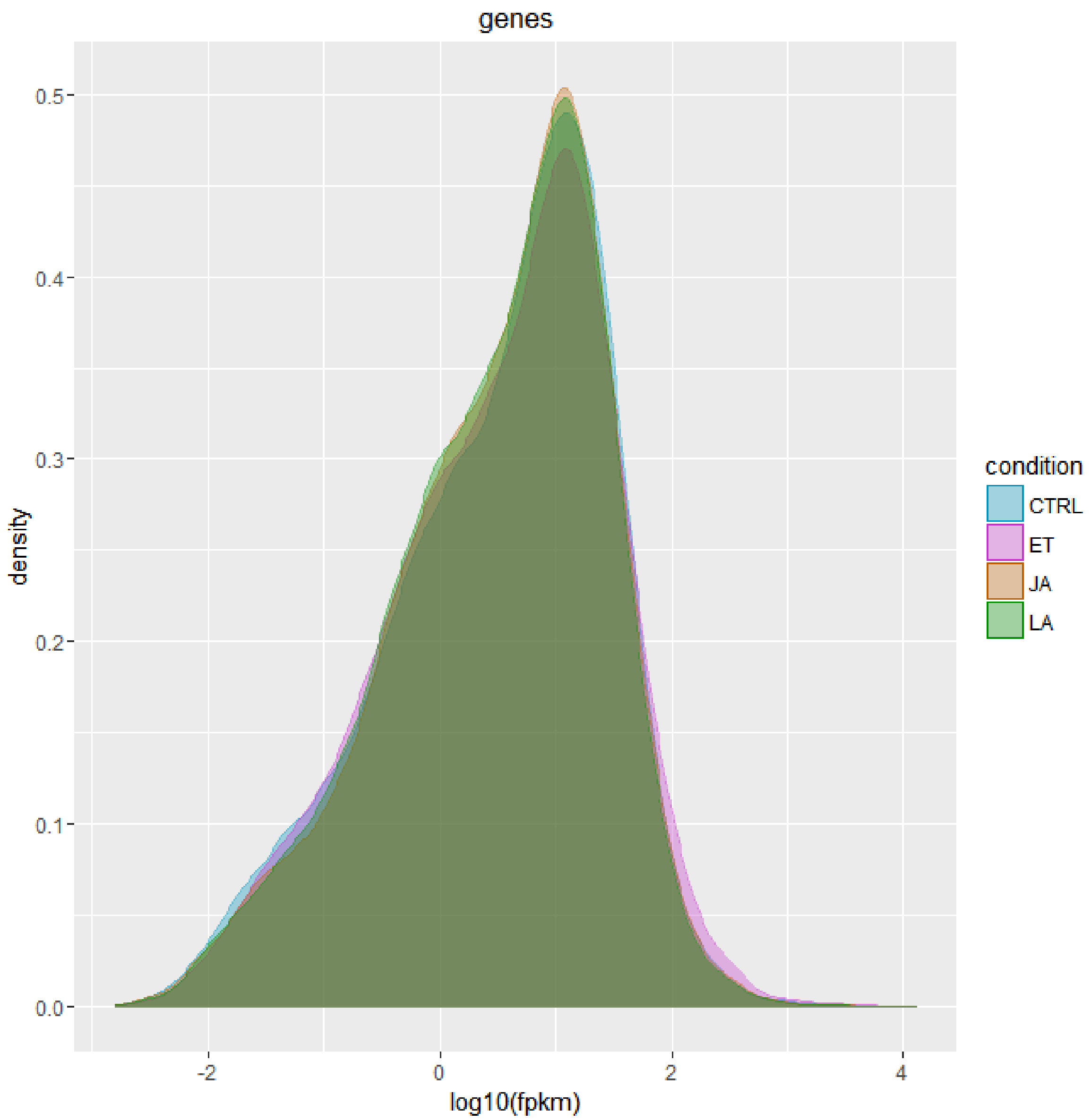

Supplementary Figure 2a. Density plot of CuffDiff data at gene level using R Bioconductor v3.2.3 (CummeRbund v2.12.1) across the samples.

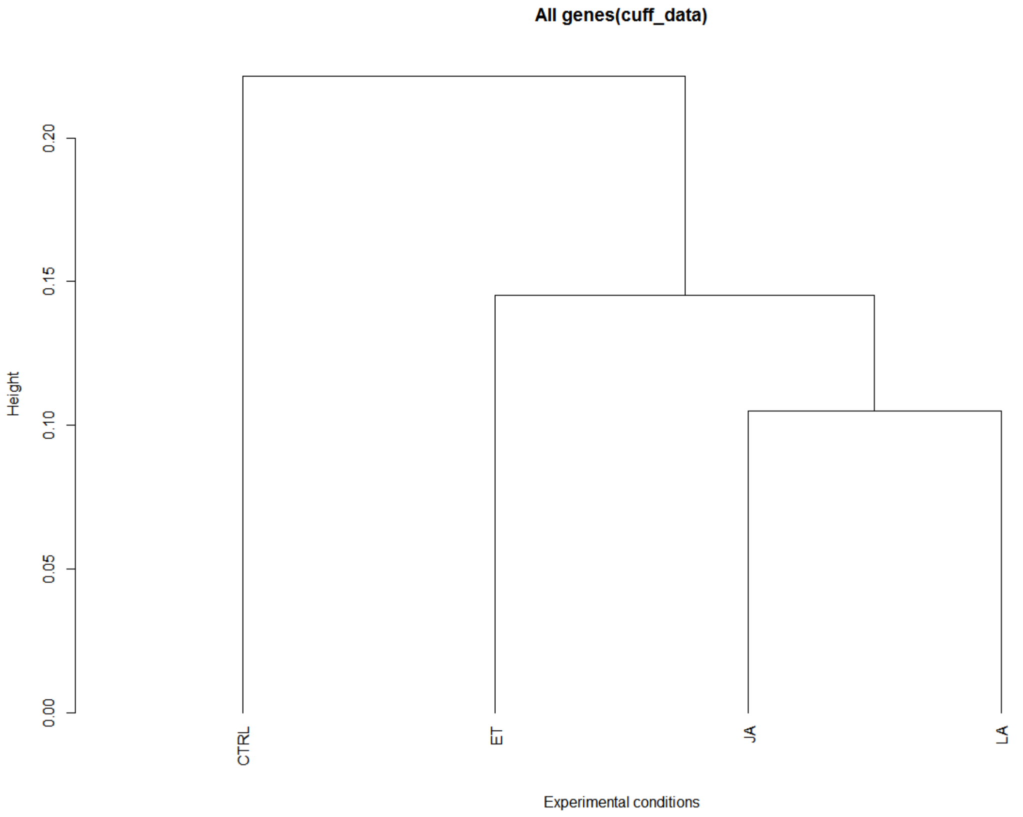

Supplementary Figure 2b. Dendrogram of CuffDiff data at gene level using R Bioconductor v3.2.3 (CummeRbund v2.12.1) across the samples.

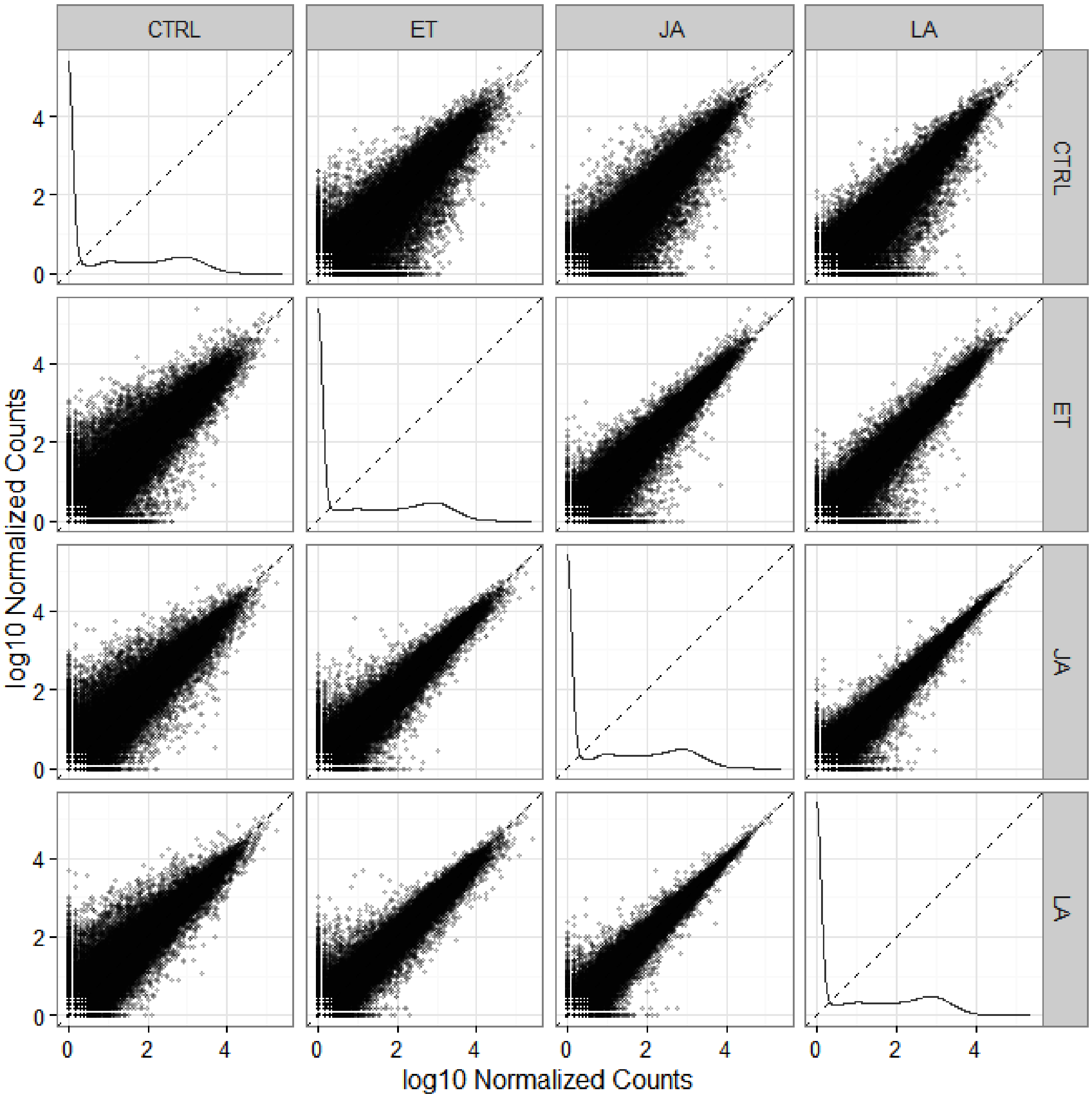

Supplementary Figure 2c. Scatter matrix plot of CuffDiff data at gene level using R Bioconductor v3.2.3 (CummeRbund v2.12.1) across the samples.

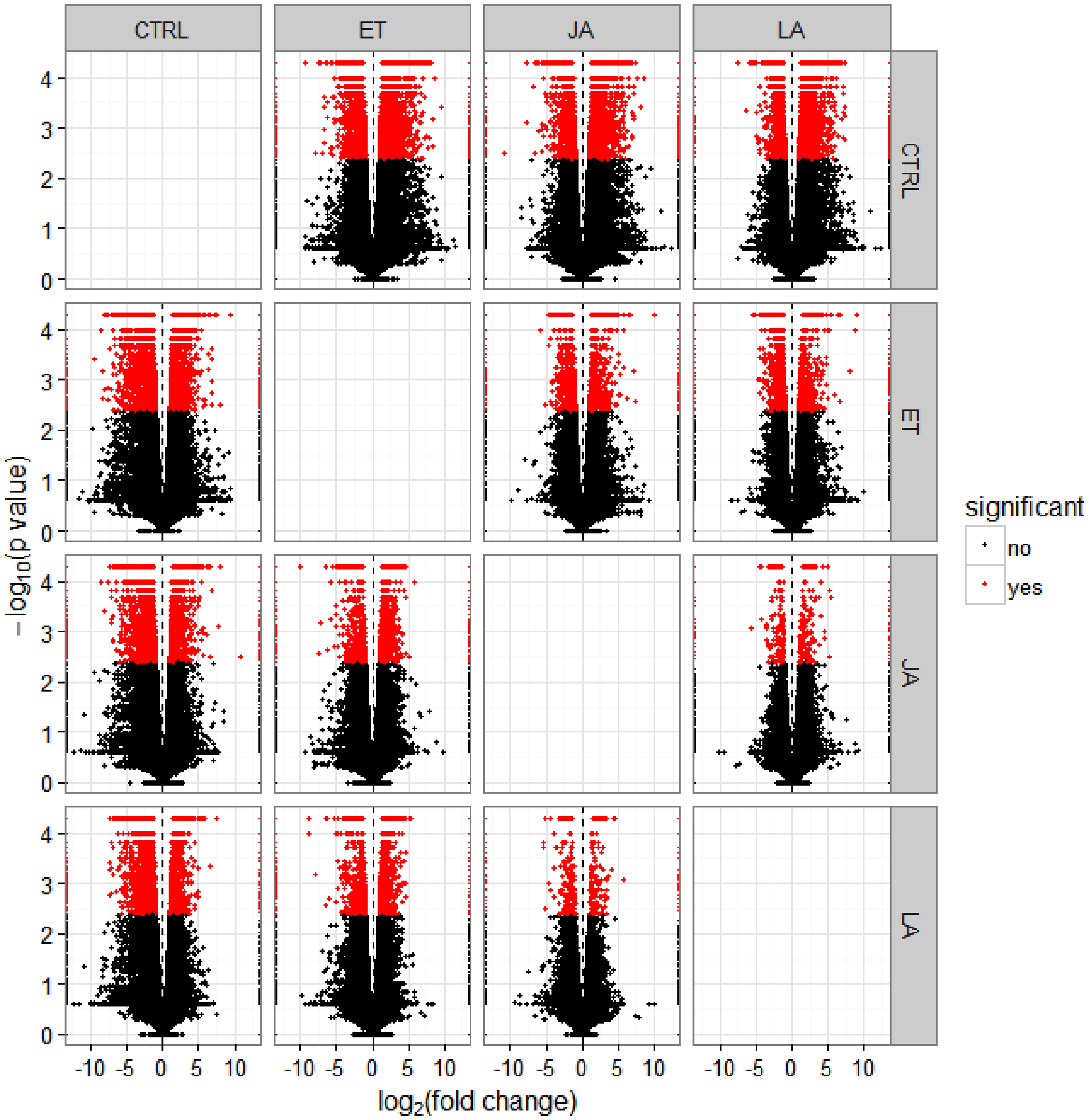

Supplementary Figure 2d. Volcano matrix plot of CuffDiff data at gene level using R Bioconductor v3.2.3 (CummeRbund v2.12.1) across the samples.

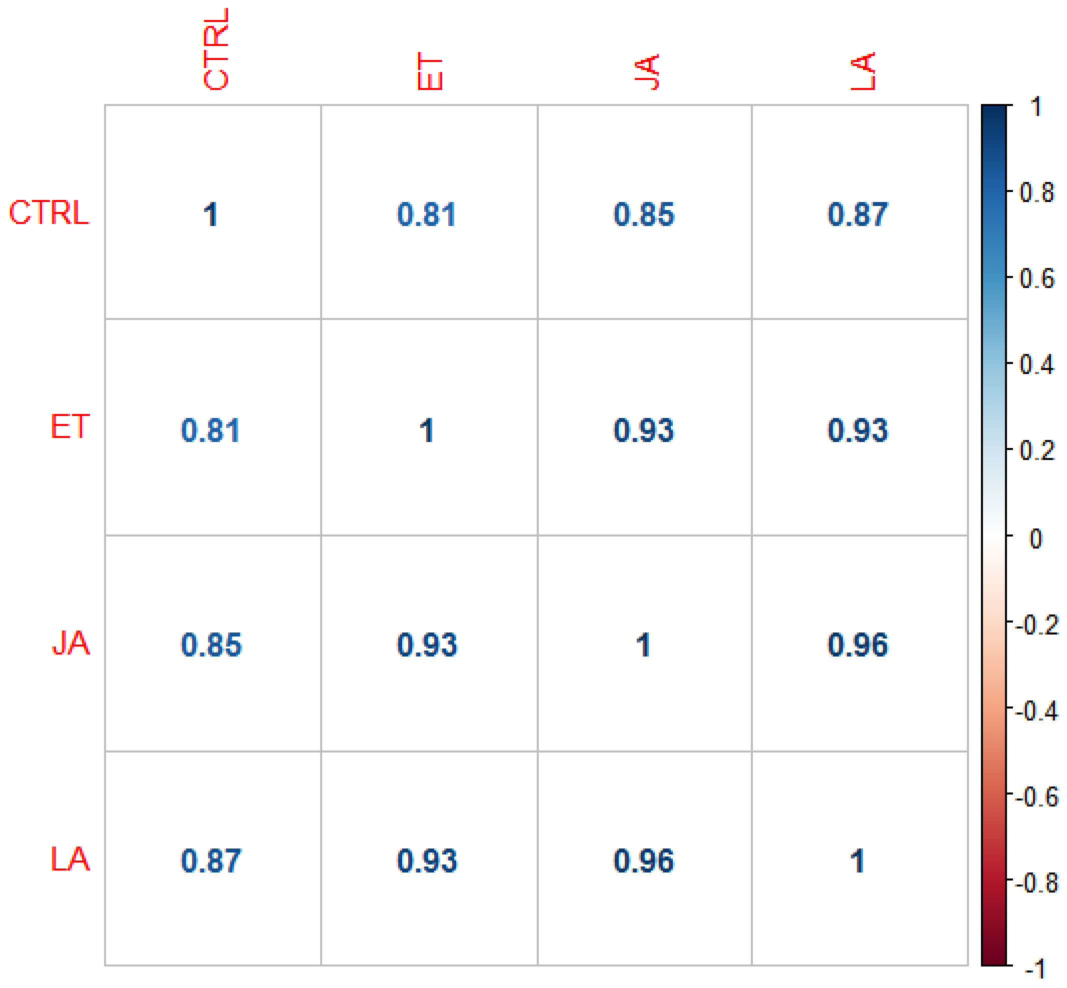

Supplementary Figure 2e. Correlation plot of CuffDiff data at gene level using R Bioconductor v3.2.3 (Corrplot v0.73) is presented in numbers across the samples.

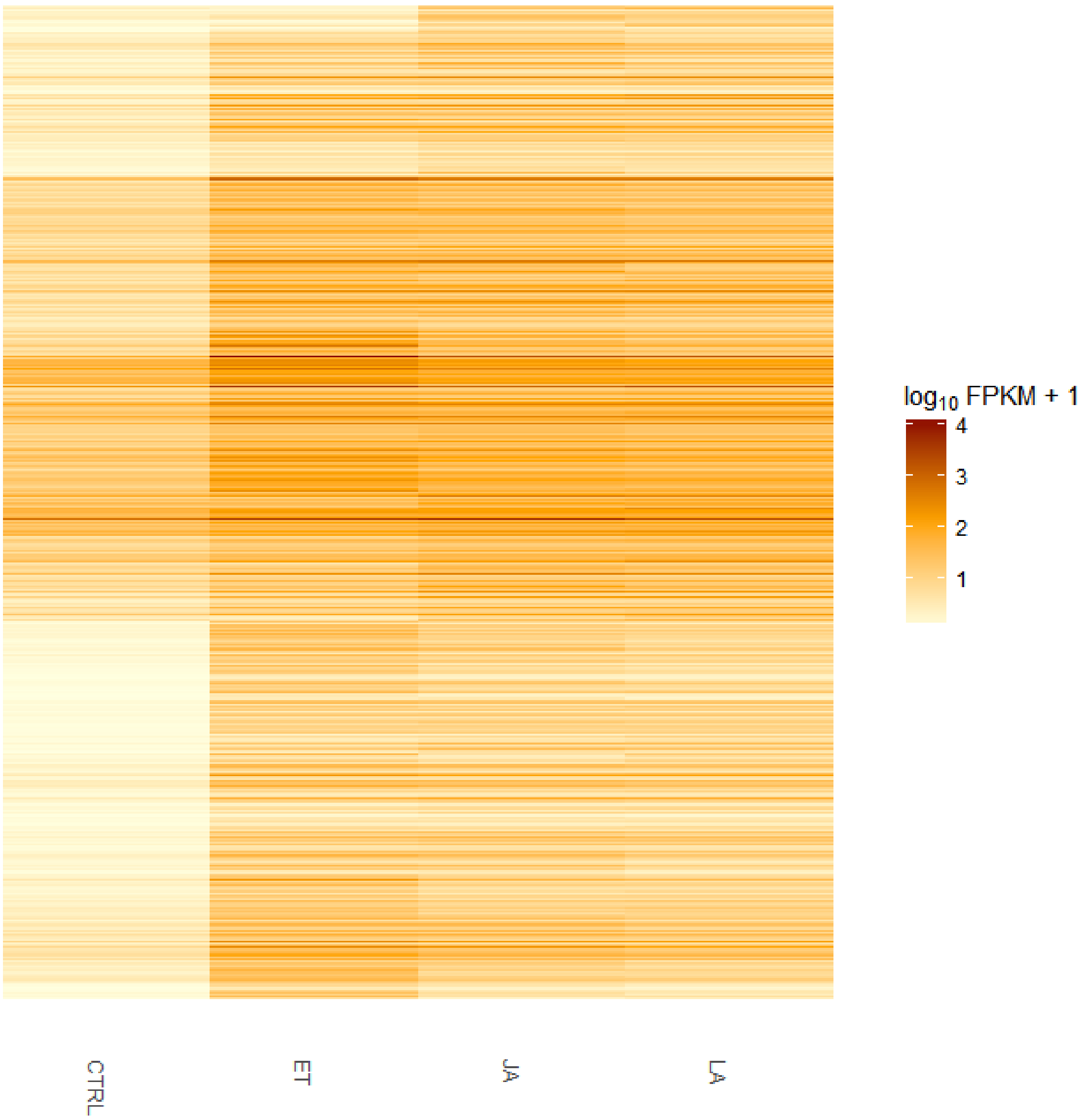

Supplementary Figure 3a. The gene expression profile of sense transcripts in JALAUP dataset presented in heatmap among the samples.

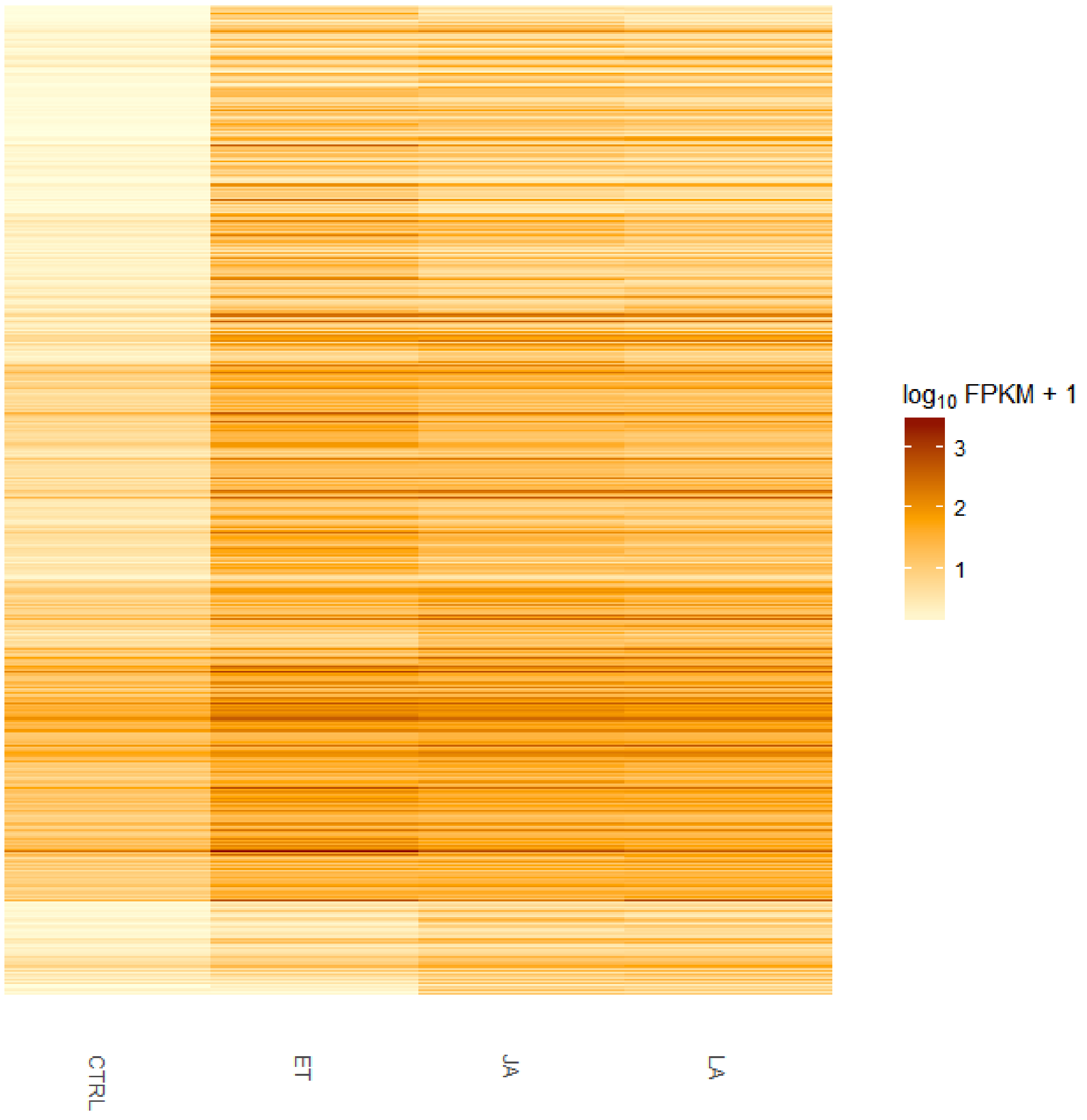

Supplementary Figure 3b. The gene expression profile of antisense transcripts in JALAUP dataset presented in heatmap among the samples.

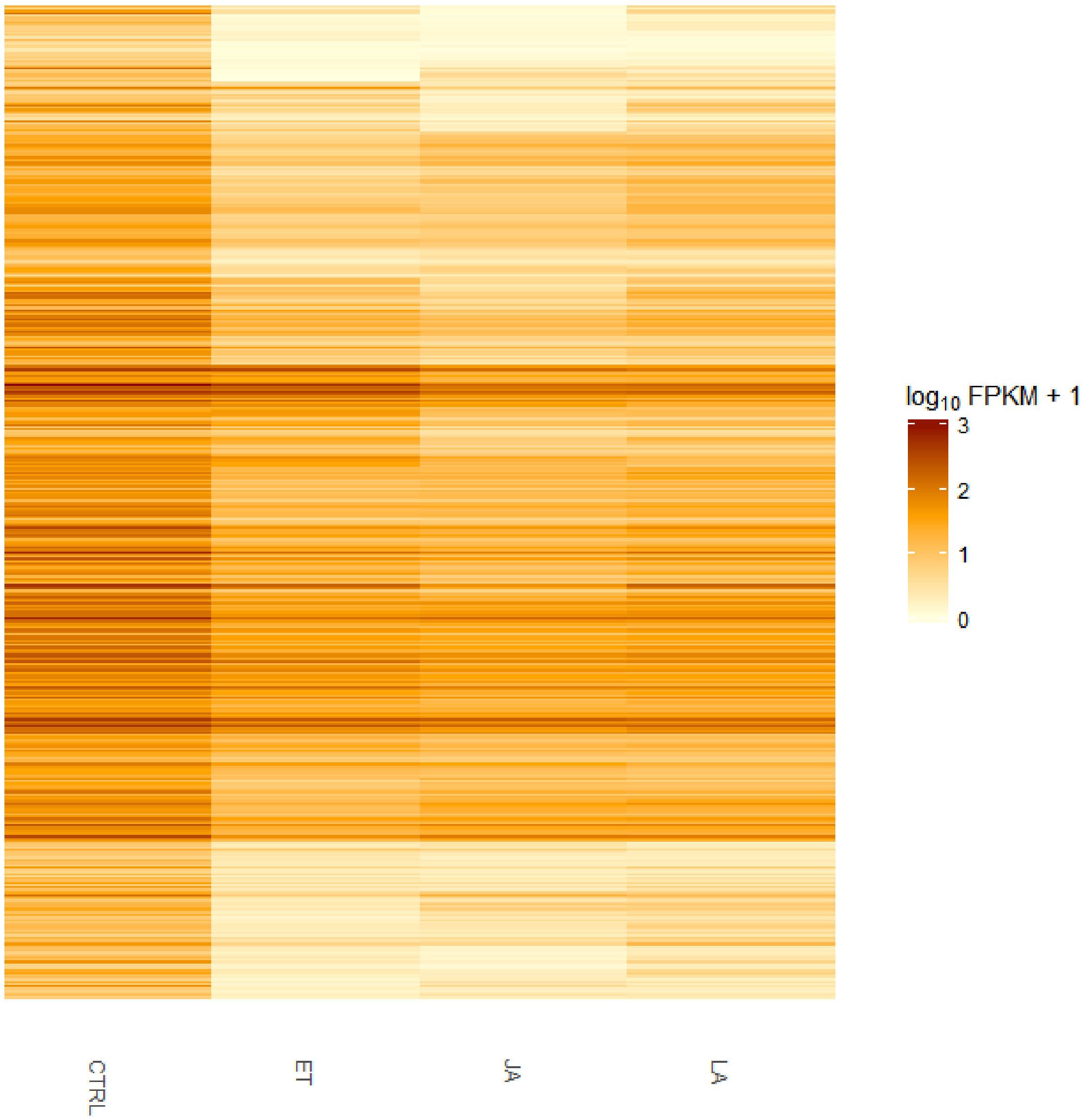

Supplementary Figure 3c. The gene expression profile of sense transcripts in JALADO dataset presented in heatmap among the samples.

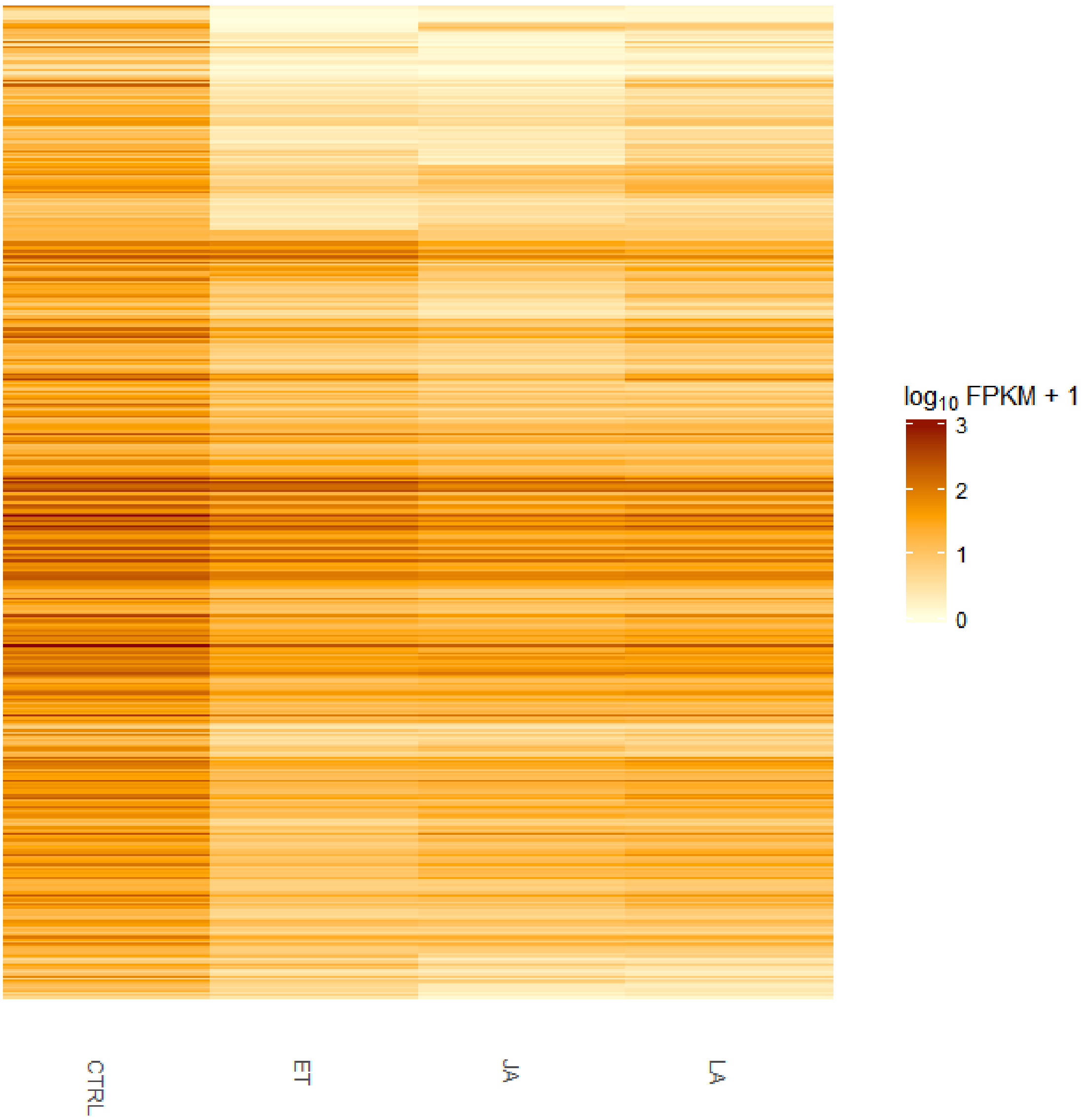

Supplementary Figure 3d. The gene expression profile of antisense transcripts in JALADO dataset presented in heatmap among the samples.

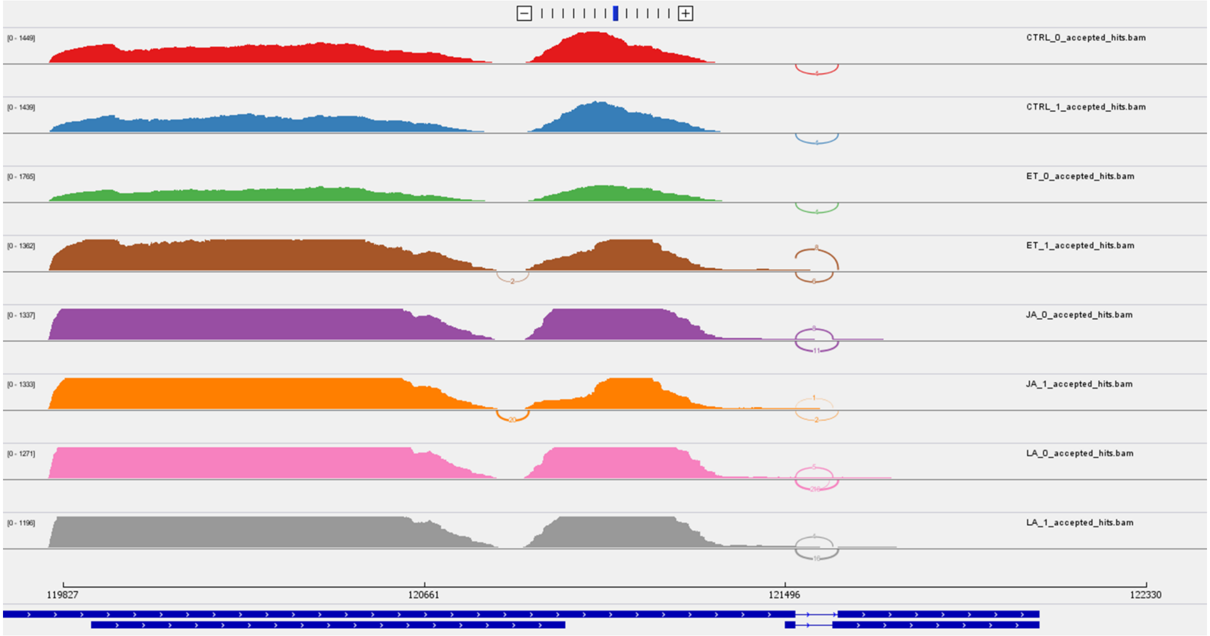

Supplementary Figure 4a. Alt 3' acceptor in MYB44 gene at genomic region Contig1950:119689-122083.

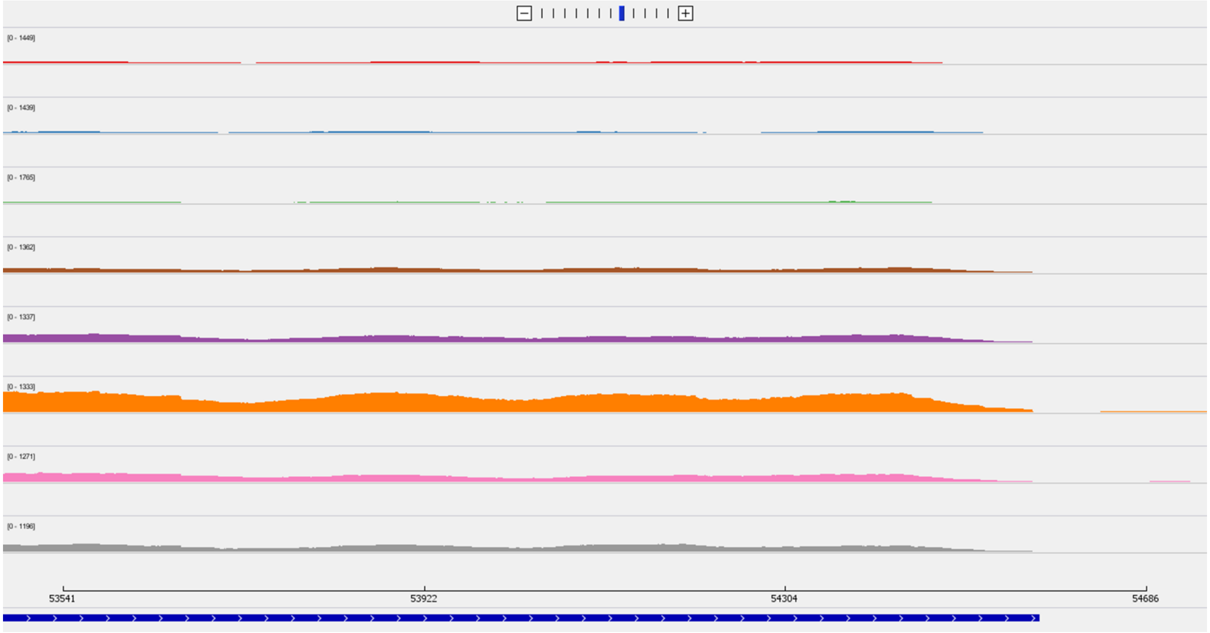

Supplementary Figure 4b. No AS event was detected in RAV1 gene at genomic region Contig33363:53478-54574.

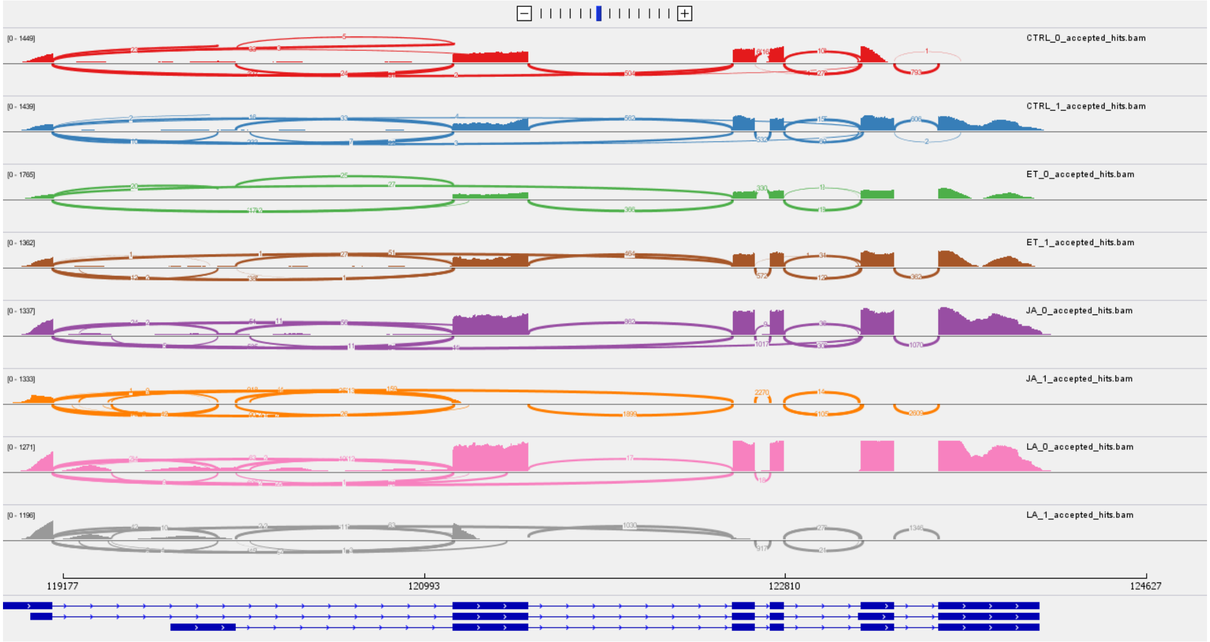

Supplementary Figure 4c. Alt 3' acceptor in NFYA3-like splice variants at genomic region Contig3656:118876-124089.

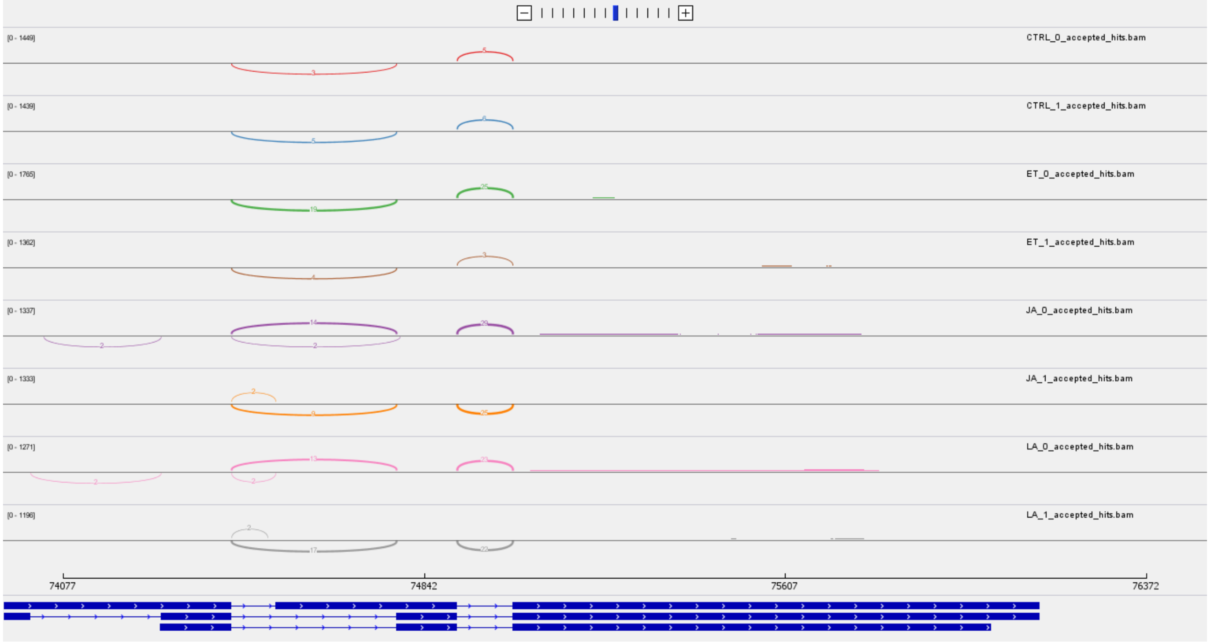

Supplementary Figure 4d. Alt 3' acceptor in MYB86 spliced variants at genomic region Contig4055:73951-76146.

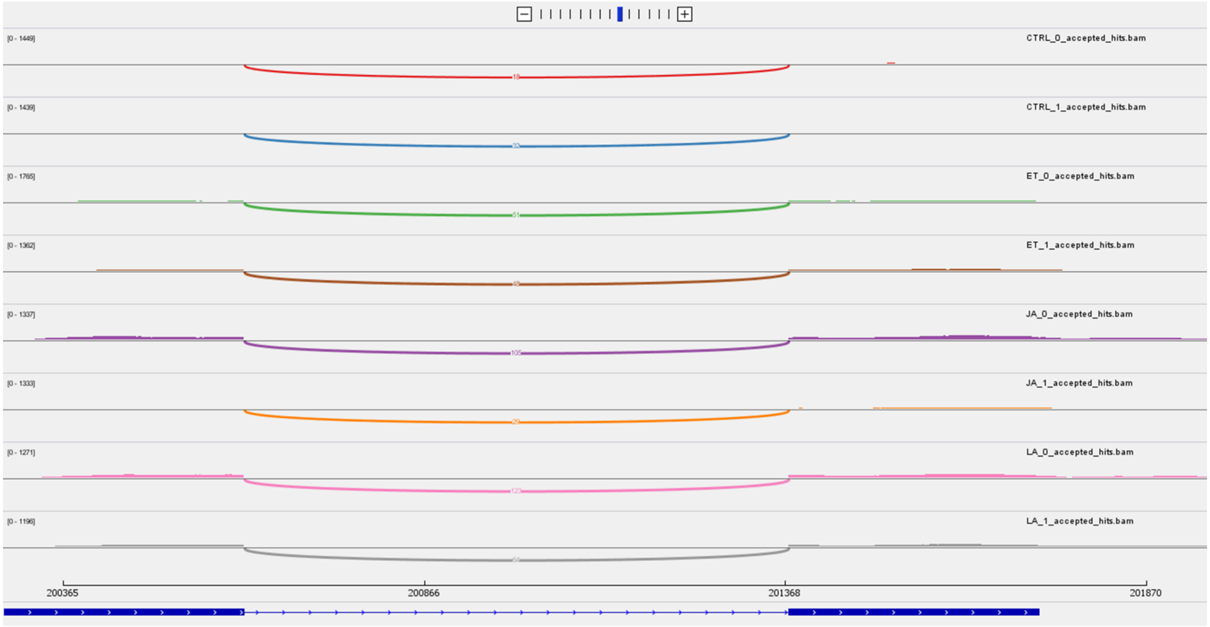

Supplementary Figure 4e. No AS event was detected in LBD1 gene at genomic region Contig421:200283-201722.

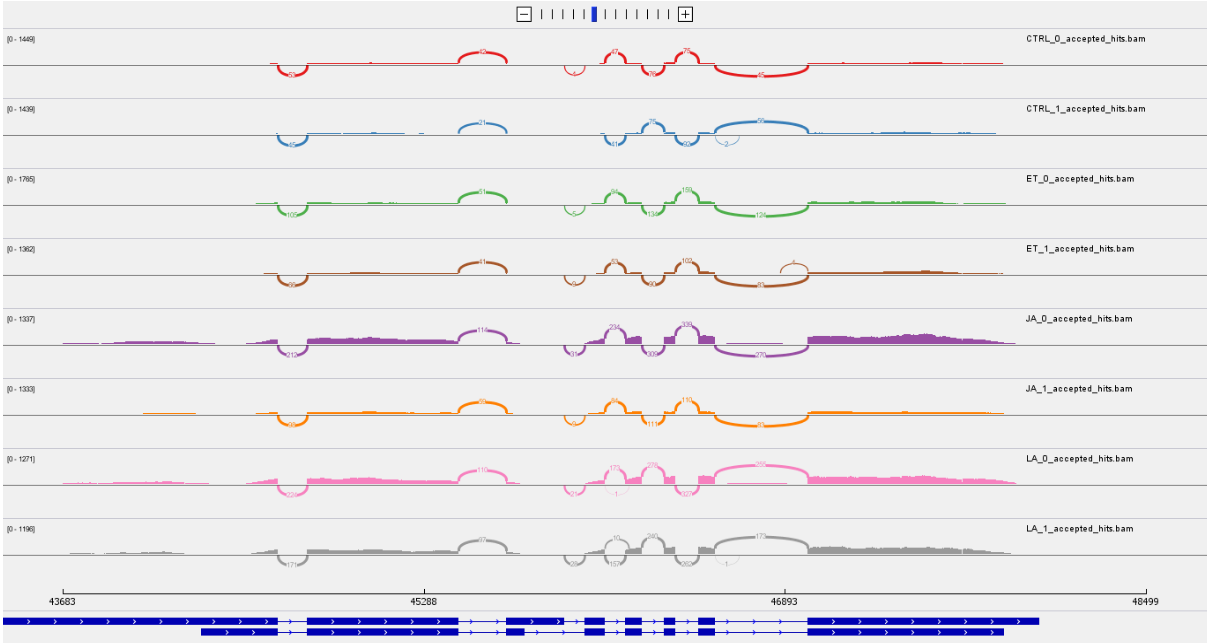

Supplementary Figure 4f. Alt 5’ donor in AP2-like spliced variants at genomic region Contig4286:43417-48023.

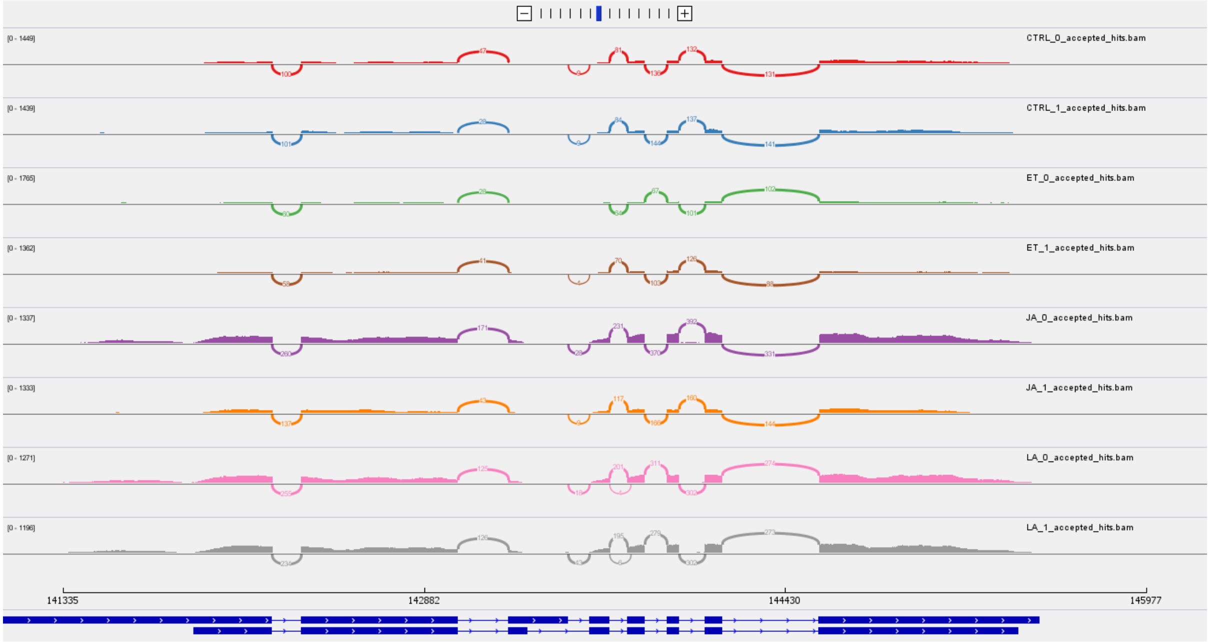

Supplementary Figure 4g. Alt 5' donor in AP2-like spliced variants at genomic region Contig5725:141079-145519.

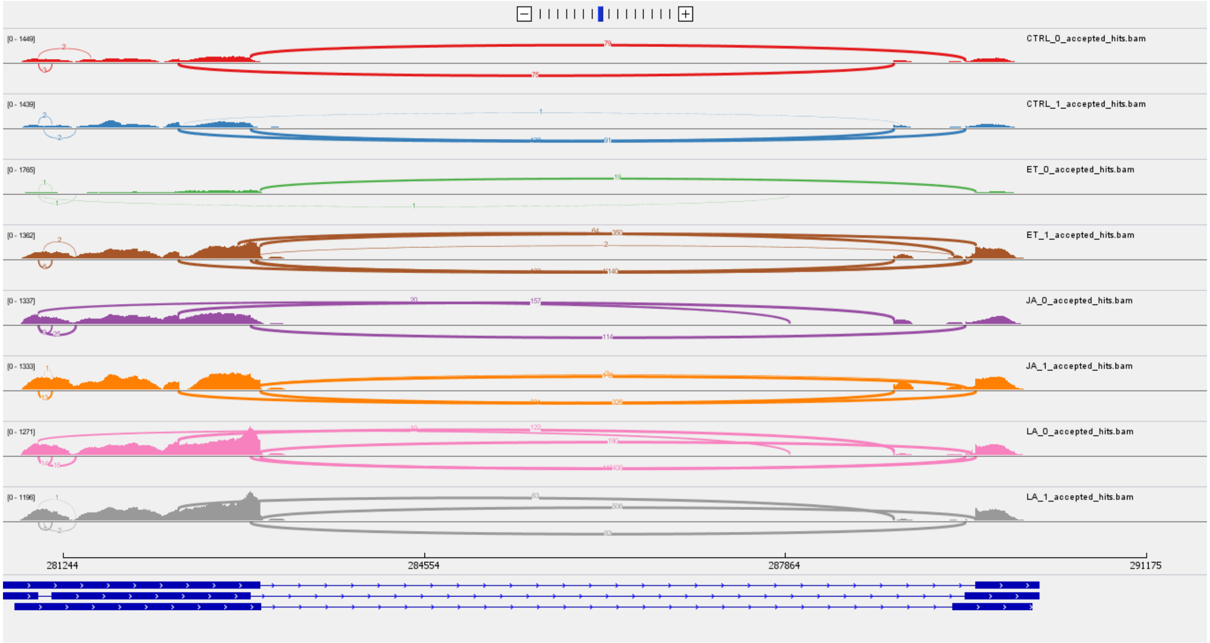

Supplementary Figure 4h. Multiple AS events in bHLH13-like spliced variants at genomic region Contig69:280694-290193.

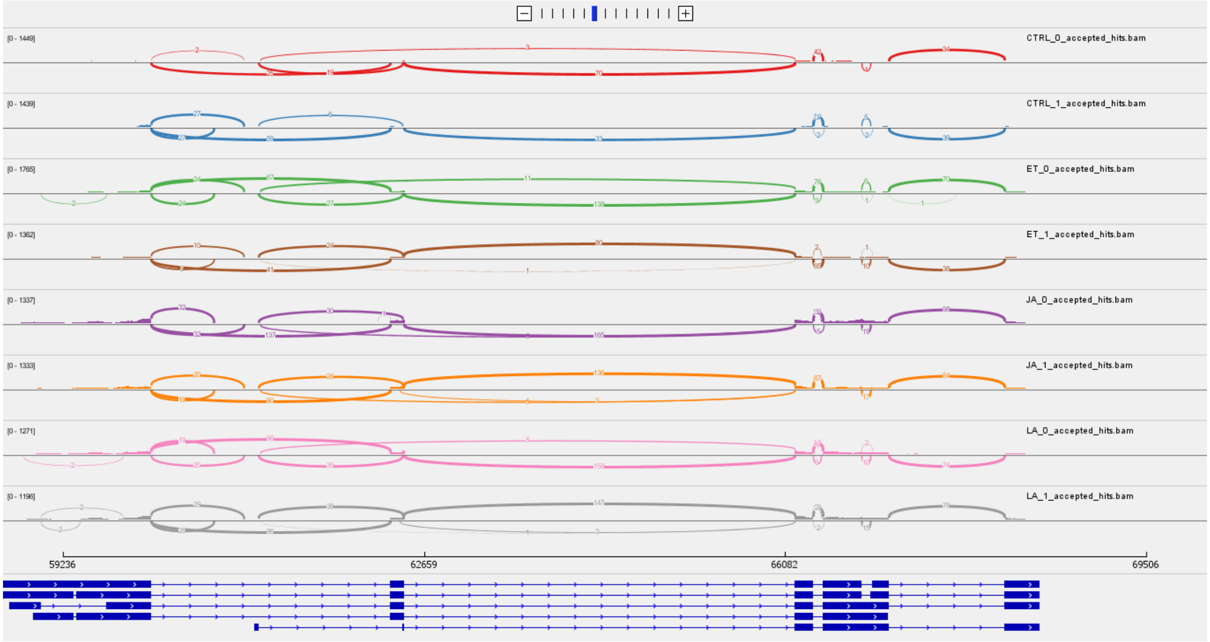

Supplementary Figure 4i. Alt acceptor, intron retention, and other AS events in SBP6 spliced variants at genomic region Contig776:58667-68490.

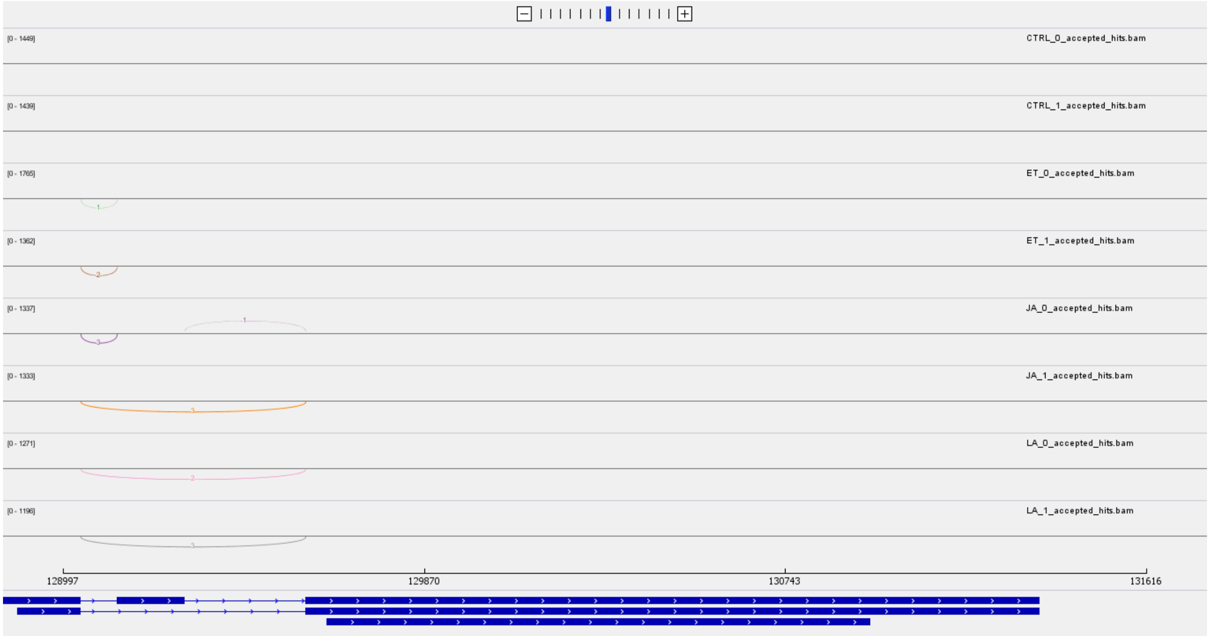

Supplementary Figure 4j. No AS event was detected in ICE1 gene at genomic region Contig19:53140-57279.

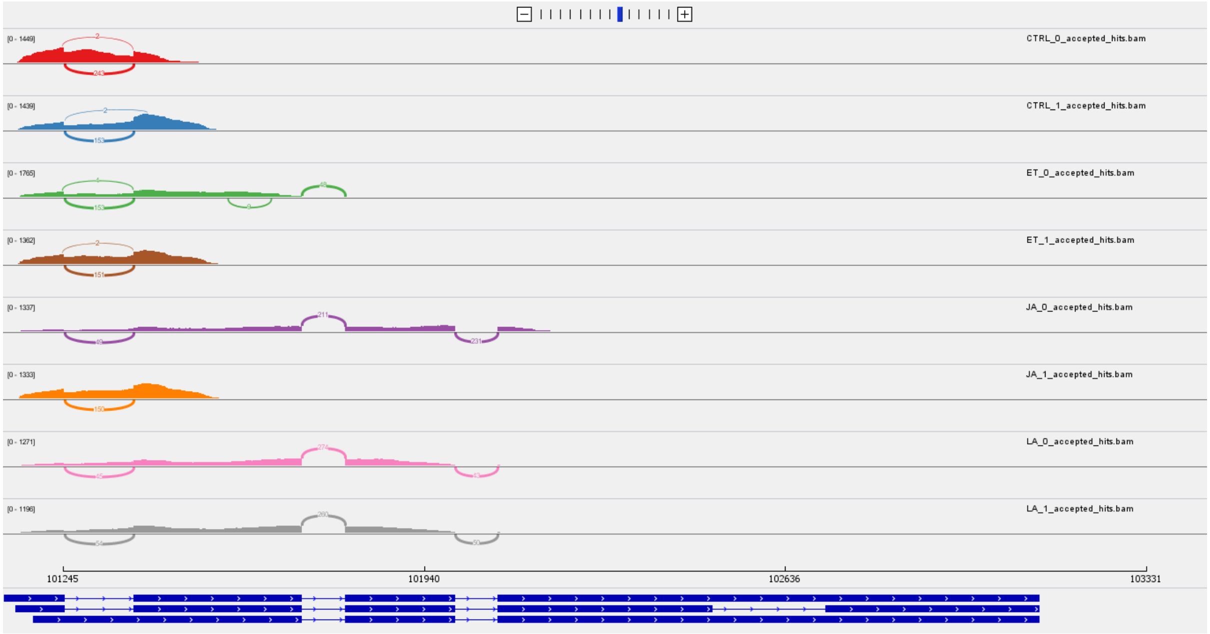

Supplementary Figure 4k. Intron retention in MYB1R1 spliced variants at genomic region Contig2073:101131-103126.

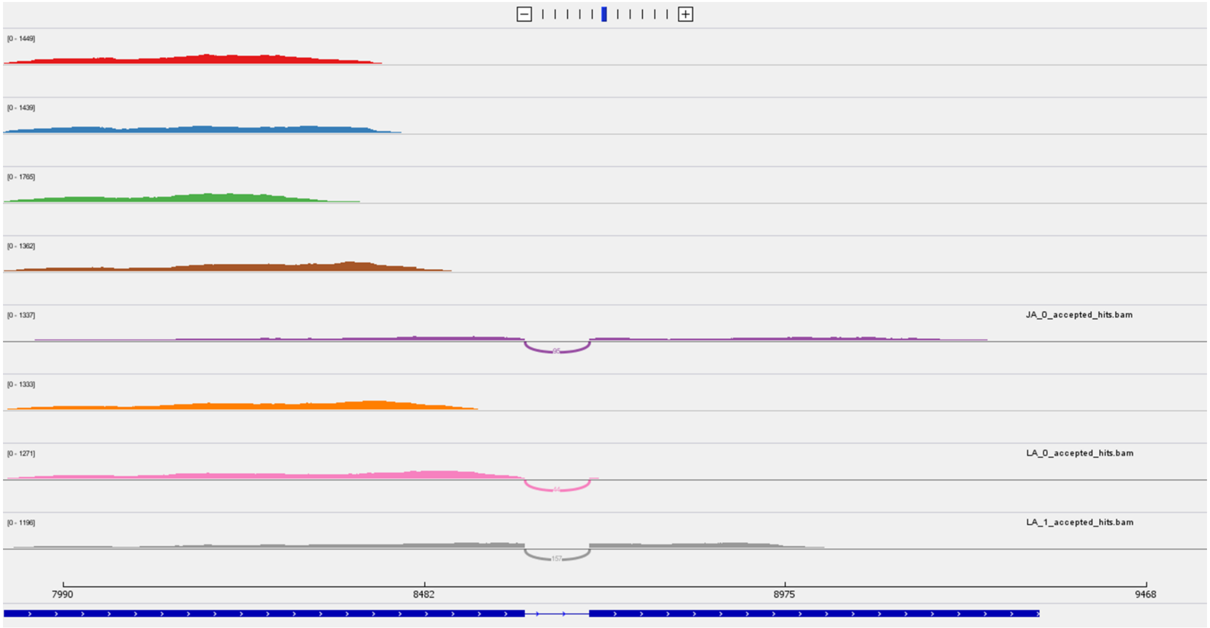

Supplementary Figure 4l. No AS event was detected in COL gene at genomic region Contig8248:7909-9322.

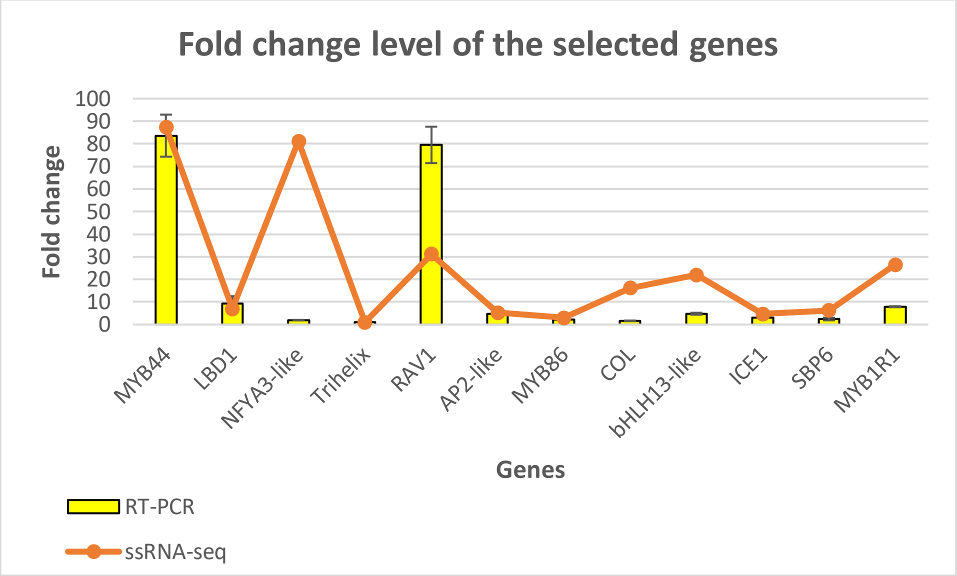

Supplementary Figure 5. Relative qPCR validation of the RNA-seq expression results of selected genes in JA-treated *Hevea* bark samples. The fold change levels for trihelix gene are 1. Each cap on the bar chart represents mean ± SEM of relative qPCR runs.

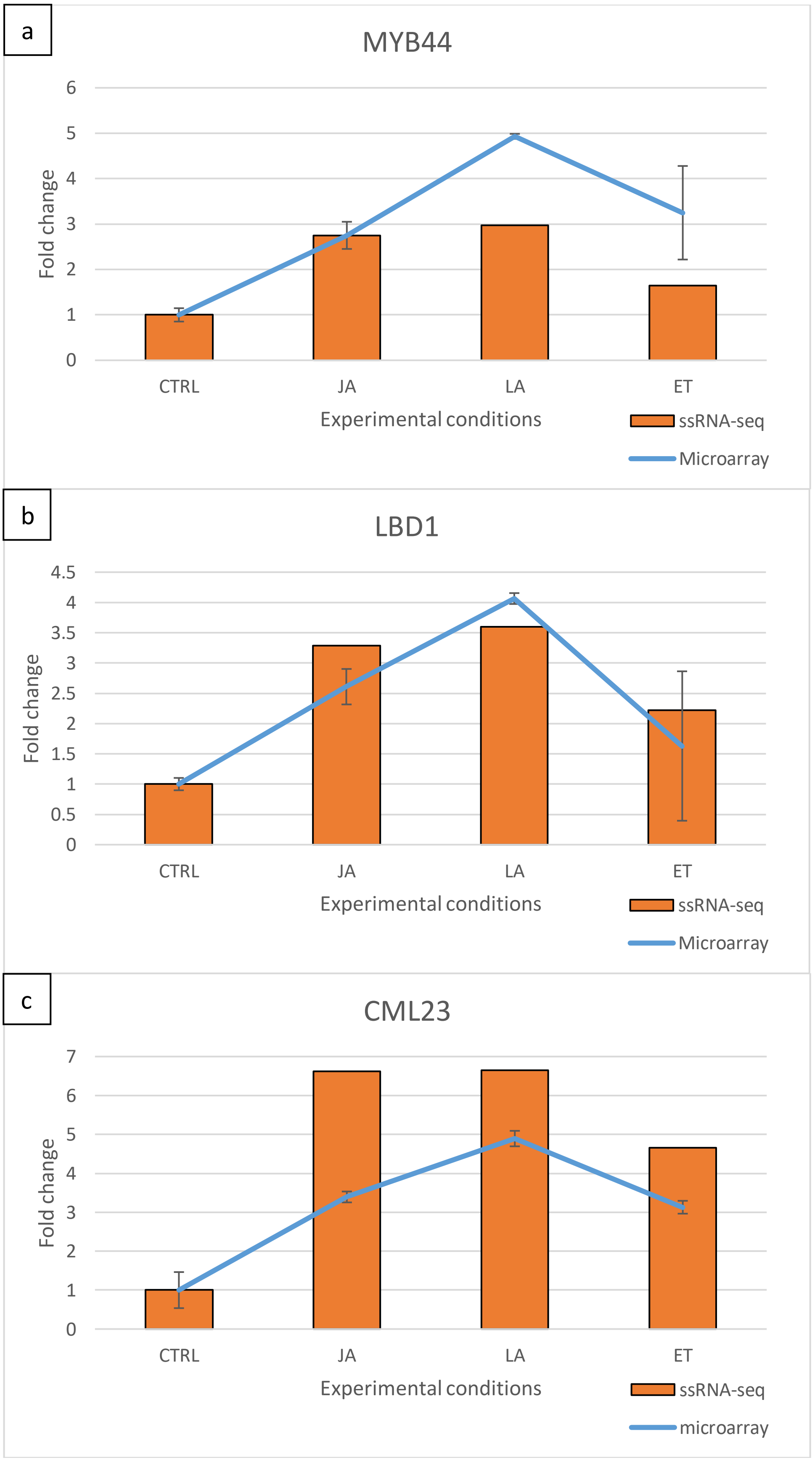

a
MYB44
Fold change
6
5
4
3
2
1
0
CTRL
JA
LA
ET
Experimental conditions
ssRNA-seq
Microarray
b
LBD1
Fold change
4.5
4
3.5
3
2.5
2
1.5
1
0.5
0
CTRL
JA
LA
ET
Experimental conditions
ssRNA-seq
Microarray
c
CML23
Fold change
7
6
5
4
3
2
1
0
CTRL
JA
LA
ET
Experimental conditions
ssRNA-seq
microarray

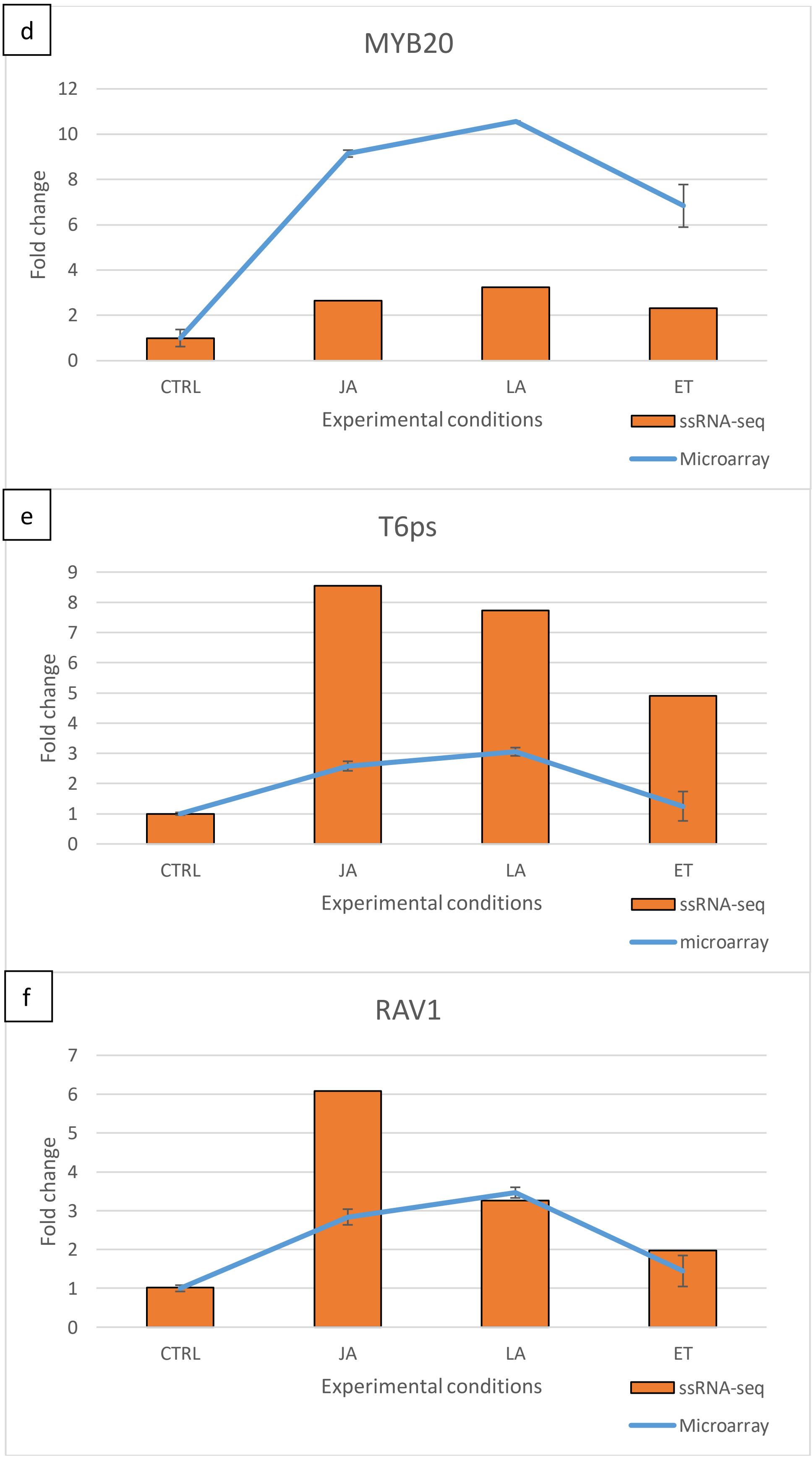
d
MYB20
12
10
8
6
4
2
0
Fold change
CTRL
JA
LA
ET
Experimental conditions
ssRNA-seq
Microarray
e
T6ps
9
8
7
6
5
4
3
2
1
0
Fold change
CTRL
JA
LA
ET
Experimental conditions
ssRNA-seq
microarray
f
RAV1
7
6
5
4
3
2
1
0
Fold change
CTRL
JA
LA
ET
Experimental conditions
ssRNA-seq
Microarray

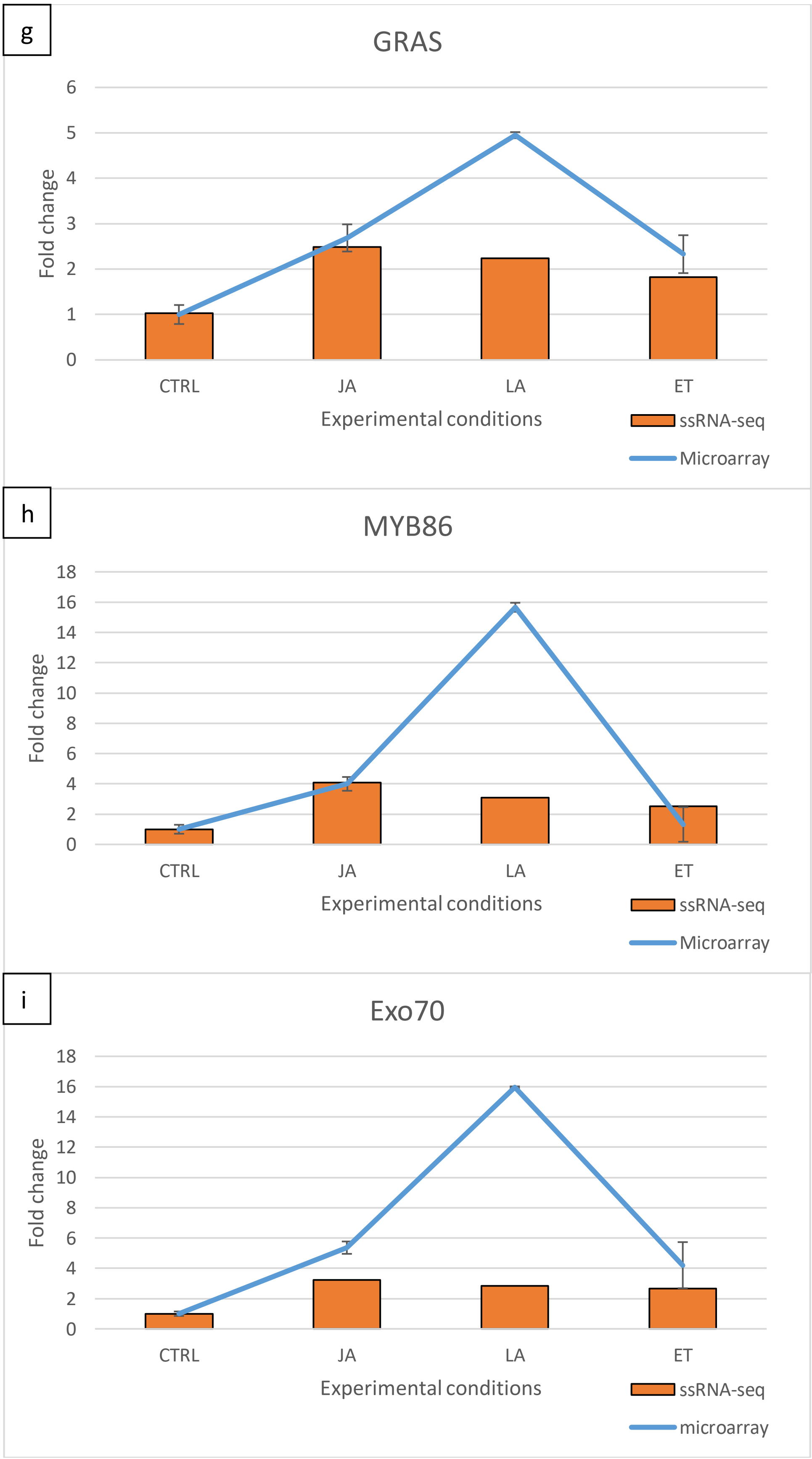

g
GRAS
Fold change
0 1 2 3 4 5 6
CTRL JA LA ET
Experimental conditions
ssRNA-seq
Microarray
h
MYB86
Fold change
0 2 4 6 8 10 12 14 16 18
CTRL JA LA ET
Experimental conditions
ssRNA-seq
Microarray
i
Exo70
Fold change
0 2 4 6 8 10 12 14 16 18
CTRL JA LA ET
Experimental conditions
ssRNA-seq
microarray

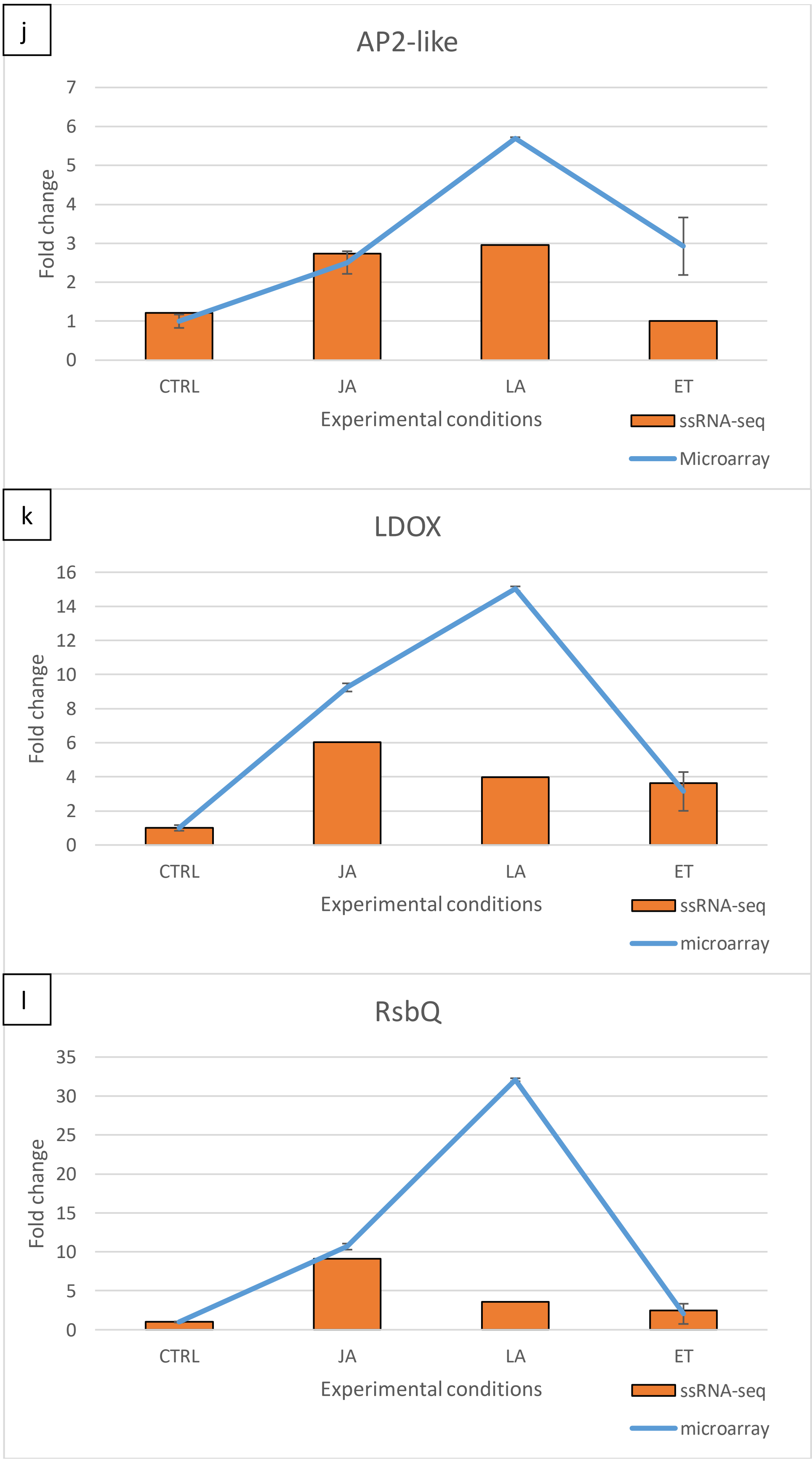
j
AP2-like
Fold change
7
6
5
4
3
2
1
0
CTRL
JA
LA
ET
Experimental conditions
ssRNA-seq
Microarray
k
LDOX
Fold change
16
14
12
10
8
6
4
2
0
CTRL
JA
LA
ET
Experimental conditions
ssRNA-seq
microarray
l
RsbQ
Fold change
35
30
25
20
15
10
5
0
CTRL
JA
LA
ET
Experimental conditions
ssRNA-seq
microarray

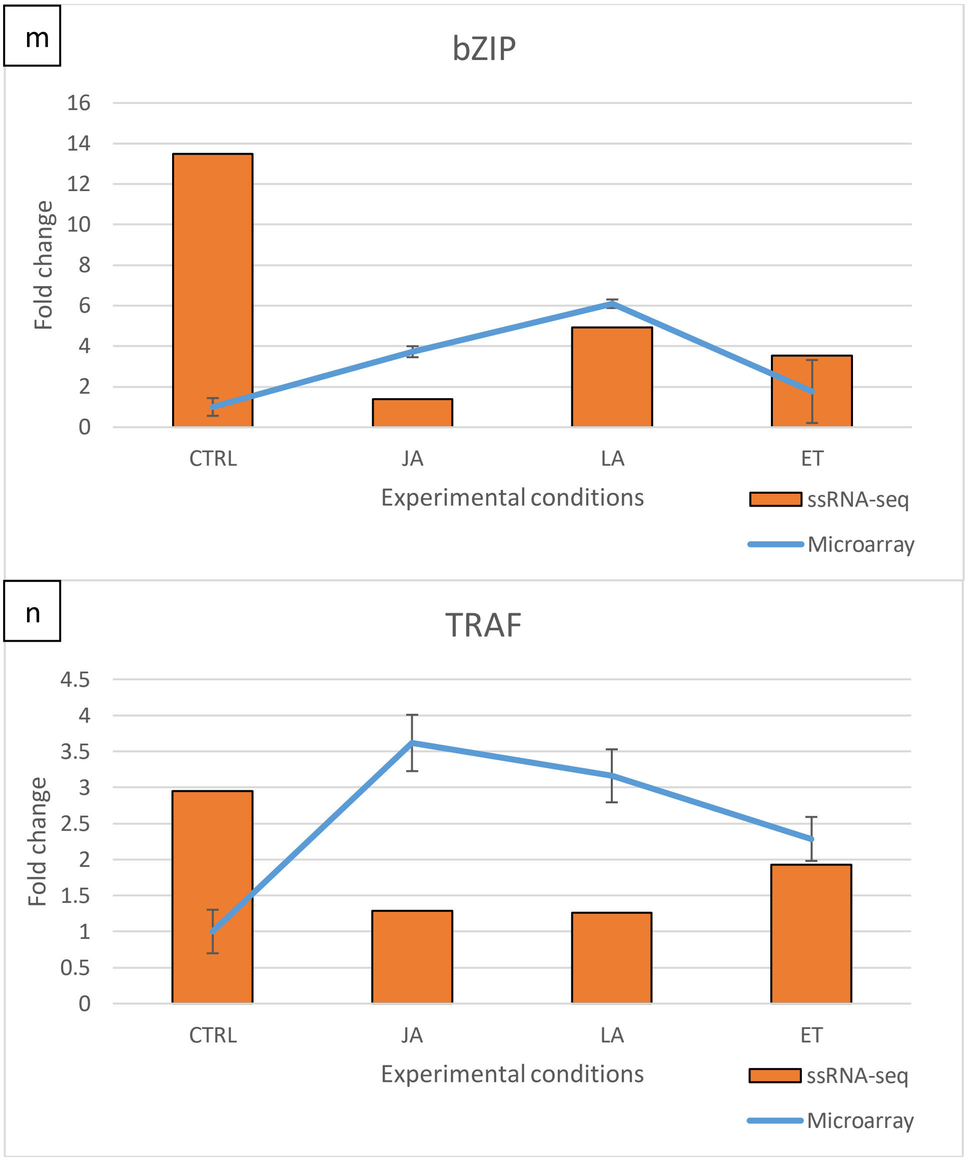

Supplementary Figure 6. Microarray validation[1] of the RNA-sequencing (RNA-seq) expression results of fourteen selected genes. (a - l) Relative mean fold change of twelve (85.71%) selected genes in RNA-seq followed the fold change pattern in the microarray data[1] where the fold change of JA- and LA-treated samples are higher than ET-treated and control samples (m and n) Relative mean fold change of two randomly selected genes did not follow the pattern in the RNA-seq data. The bar chart indicates values from RNA-seq while line chart indicates values from the previous microarray study[1]. Each cap on the line chart represents mean ± SEM of relative expression of microarray study.

Supplementary Table 1. The raw sequence reads information.

Sample	Total Bases	Read Count	GC (%)	Q20 (%)
CTRL_0	15,651,897,752	103,654,952	43.86	97.03
CTRL_1	15,211,050,232	100,735,432	44.34	97.42
ET_0	16,004,452,854	105,989,754	45.05	97
ET_1	14,989,054,562	99,265,262	44.09	97.22
JA_0	14,719,921,222	97,482,922	43.87	95.75
JA_1	15,278,812,992	101,184,192	43.74	95.84
LA_0	14,886,763,840	98,587,840	43.75	96.28
LA_1	14,135,784,668	93,614,468	43.91	95.36

Supplementary Table 2. The strand information in each sample and dataset

Sample	Unknown	Antisense transcripts	Sense transcripts	Total
CTRL_0	5	29,174 (49.90%)	29,062 (50.09%)	58,241
CTRL_1	2	29,495 (49.86%)	29,330 (50.14%)	58,827
ET_0	1	25,700 (49.80%)	25,496 (50.20%)	51,197
ET_1	6	29,495 (49.93%)	29,416 (50.06%)	58,917
JA_0	1	29,558 (49.85%)	29,383 (50.15%)	58,942
JA_1	3	29,558 (50.08%)	29,652 (49.92%)	59,213
LA_0	2	30,041 (50.08%)	30,145 (49.91%)	60,188
LA_1	3	29,336 (49.91%)	29,238 (50.08%)	58,577

Dataset	Unknown	Antisense transcripts	Sense transcripts	Total
JALAUP (Fig. 3A)	0	879 (50.72%)	854 (49.28%)	1,733
JALADO (Fig. 3B)	0	480 (51.56%)	451 (48.44%)	931
FUP (Fig. 3C)	0	124 (52.32%)	113 (47.68%)	237
FDO (Fig. 3D)	0	111 (52.11%)	102 (47.89%)	213

Supplementary Table 5. Gene prediction for the unknown, intergenic transcripts in the JALA dataset (class code ‘u’). Asterisk indicates genomic region in FDE dataset.

	Sequence name	Hit name
*	Contig104481_3_488_+	gi|255568633|ref|XP_002525290.1|transferase, putative
	Contig130015_1683_2392_+	gi|802733806|ref|XP_012086699.1|PREDICTED: glutaredoxin domain-containing cysteine-rich protein 1
	Contig130901_73_587_+	gi|353678016|sp|A8CDT2.1|BAS_BRUGY
	Contig141340_253_1632_-	gi|255541166|ref|XP_002511647.1|DNA binding protein, putative
	Contig155570_264_901_-	gi|802550675|ref|XP_012093109.1|PREDICTED: ATP-dependent Clp protease proteolytic subunit-related protein 1, chloroplastic
	Contig156357_3975_5834_+	gi|748015737|gb|AJE29370.1|putative gag protein
	Contig246_251547_254202_-	gi|802570322|ref|XP_012068116.1|PREDICTED: xylulose kinase
	Contig246_251547_254259_-	gi|802570322|ref|XP_012068116.1|PREDICTED: xylulose kinase
	Contig246_251547_254260_-	gi|802570322|ref|XP_012068116.1|PREDICTED: xylulose kinase
	Contig2626_55057_58813_-	gi|18378613|gb|AAL68644.1|AF458768_1polyprotein
	Contig2759_175638_176063_-	gi|802787874|ref|XP_012092034.1|PREDICTED: UDP-glycosyltransferase 74B1
*	Contig3751_114385_117355_-	gi|568519779|ref|YP_008992013.1|polyprotein
	Contig43298_1_668_+	gi|255543106|ref|XP_002512616.1|PREDICTED: dynamin-related protein 4C
	Contig58257_1256_1738_+	gi|255558031|ref|XP_002520044.1|PREDICTED: bark storage protein A
	Contig594_115418_121714_+	gi|400131576|emb|CCH50976.1|T4.15
	Contig68611_1_1232_+	gi|113205323|gb|AAT38747.2|Polyprotein, putative
	Contig78594_1_603_-	gi|802746038|ref|XP_012087499.1|PREDICTED: O-acyltransferase WSD1-like
	Contig9680_42064_42486_+	gi|255543106|ref|XP_002512616.1|PREDICTED: dynamin-related protein 4C
	Contig103140_3020_3422_+	gi|464707|sp|P34788.1|RS18_ARATH
*	Contig142381_4_2405_+	gi|255567007|ref|XP_002524486.1|alcohol dehydrogenase, putative
	Contig165267_364_1014_-	gi|802766205|ref|XP_012090081.1|PREDICTED: glutamate synthase 1
	Contig179_75733_79317_+	gi|20141760|sp|P49215.3|RS17_SOLLC
	Contig179_76018_79317_+	gi|20141760|sp|P49215.3|RS17_SOLLC
	Contig179_77476_79317_+	gi|20141760|sp|P49215.3|RS17_SOLLC

	Contig179_78465_79248_+	gi|20141760|sp|P49215.3|RS17_SOLLC
*	Contig185024_4_645_-	gi|255564962|ref|XP_002523474.1|TMV resistance protein N, putative
*	Contig1956_84574_87585_+	gi|255586180|ref|XP_002533749.1|transcription factor, putative
*	Contig1956_84575_87585_+	gi|255586180|ref|XP_002533749.1|transcription factor, putative
	Contig247_17476_18232_+	gi|2586082|gb|AAB82754.1|retrofit
*	Contig270_322060_323664_-	gi|255540813|ref|XP_002511471.1|two-component sensor protein histidine protein kinase, putative
*	Contig270_322060_327591_-	gi|255540813|ref|XP_002511471.1|two-component sensor protein histidine protein kinase, putative
*	Contig37960_1_3038_+	gi|130582|sp|P10978.1|POLX_TOBACRecName: Full=Retrovirus-related Pol polyprotein from transposon TNT 1-94; Includes: RecName: Full=Protease; Includes: RecName: Full=Reverse transcriptase; Includes: RecName: Full=Endonuclease
*	Contig37960_3117_4122_+	gi|342365809|gb|AEL30343.1|RNA-directed DNA polymerase
	Contig3881_117580_120134_-	gi|802588622|ref|XP_012070988.1|PREDICTED: serine acetyltransferase 1, chloroplastic-like
*	Contig42485_1_548_+	gi|255561558|ref|XP_002521789.1|Disease resistance protein RFL1, putative
	Contig4324_243337_244370_+	gi|566206109|ref|XP_006374316.1|chitinase family protein
	Contig5460_76367_78267_+	gi|255556177|ref|XP_002519123.1|F-box and wd40 domain protein, putative
*	Contig58202_629_1077_+	gi|802546496|ref|XP_012085165.1|PREDICTED: transcription factor bHLH62
	Contig69075_310_633_+	gi|15214148|sp|Q9C7F5.1|NTF2_ARATH
	Contig7136_17052_22364_-	gi|297848564|ref|XP_002892163.1|coproporphyrinogen III oxidase
	Contig73263_1_505_-	gi|506444379|gb|AGM14950.1|sucrose synthase 5
	Contig73263_1_566_-	gi|506444379|gb|AGM14950.1|sucrose synthase 5
*	Contig82766_1_502_-	gi|802574128|ref|XP_012068673.1|PREDICTED: protein ASPARTIC PROTEASE IN GUARD CELL 2
	Contig84896_1_1176_+	gi|802701768|ref|XP_012083978.1|PREDICTED: RING-H2 finger protein ATL2
	Contig915_58334_67125_+	gi|255579803|ref|XP_002530739.1|ATP binding protein, putative
	Contig915_58357_61923_+	gi|255579803|ref|XP_002530739.1|ATP binding protein, putative
*	Contig98092_9_498_+	gi|255566835|ref|XP_002524401.1|basic 7S globulin 2 precursor small subunit, putative
	Contig99603_8_616_+	gi|12229632|sp|O24146.1|4CL2_TOBAC

Supplementary Table 10. Identification and classification of TFs and TRs on (a) FUP (b) FDO dataset using iTAK program.

(a) Genomic location	TF/TR	Gene family->Gene subfamily
Contig1511_296069_299439_-	TF	C3H
Contig1511_296341_298366_-	TF	C3H
Contig1950_119690_122083_+	TF	MYB->MYB
Contig1950_119893_120988_+	TF	MYB->MYB
Contig1956_84574_87585_+	TF	GRAS
Contig1956_84575_87585_+	TF	GRAS
Contig2311_191895_192807_-	TF	AP2/ERF->AP2/ERF-ERF
Contig2325_55860_58099_-	TF	MYB->MYB
Contig2325_56951_58099_-	TF	MYB->MYB-related
Contig245_233924_237916_-	TF	GRAS
Contig245_234113_235742_-	TF	GRAS
Contig2641_91072_93784_-	TF	WRKY
Contig2641_92029_93742_-	TF	WRKY
Contig2677_76618_78896_+	TF	MYB->MYB
Contig2677_76904_78615_+	TF	MYB->MYB-related
Contig270_322060_323444_-	TR	Orphans
Contig270_322060_323664_-	TR	Orphans
Contig270_322060_327591_-	TR	Orphans
Contig2_539741_546443_+	TR	SNF2
Contig2_539741_546640_+	TR	SNF2
Contig30_127387_128437_-	TF	C2C2->C2C2-GATA
Contig30_127387_129100_-	TF	C2C2->C2C2-GATA
Contig329_435686_438435_-	TF	SBP
Contig329_435720_438069_-	TF	SBP
Contig33363_53479_54574_+	TF	AP2/ERF->AP2/ERF-RAV
Contig3442_4170_8027_-	TR	Orphans
Contig3656_118877_124089_+	TF	NF-Y->NF-YA
Contig3656_119015_124089_+	TF	NF-Y->NF-YA
Contig3656_119720_124089_+	TF	NF-Y->NF-YA
Contig4055_73952_76146_+	TF	MYB->MYB
Contig4055_73953_76146_+	TF	MYB->MYB
Contig4055_74284_76044_+	TF	MYB->MYB
Contig421_200284_201722_+	TF	LOB
Contig4286_43418_48023_+	TF	AP2/ERF->AP2/ERF-ERF
Contig4286_44297_47865_+	TF	AP2/ERF->AP2/ERF-AP2
Contig5306_21934_24116_-	TF	MYB->MYB
Contig5306_21934_24122_-	TF	MYB->MYB-related
Contig5306_22015_24122_-	TF	MYB->MYB
Contig5725_141080_145519_+	TF	AP2/ERF->AP2/ERF-ERF
Contig5725_141894_145428_+	TF	AP2/ERF->AP2/ERF-AP2
Contig670_60984_61848_-	TF	MYB->MYB

Contig69_280695_290193_+	TF	bHLH
Contig69_280801_290131_+	TF	bHLH
Contig735_126111_128787_+	TF	bHLH
Contig735_126111_128983_+	TF	bHLH
Contig73973_112299_112995_-	TF	AP2/ERF->AP2/ERF-ERF
Contig776_58668_68490_+	TF	SBP
Contig776_58728_68490_+	TF	SBP
Contig776_58948_67057_+	TF	SBP
Contig914_129635_130949_+	TF	Trihelix

**(b) Genomic location**	**TF/TR**	**Gene family->Gene subfamily**
Contig103_208536_216660_-	TF	Tify
Contig103_210994_216660_-	TF	Tify
Contig103_211964_216643_-	TF	Tify
Contig1080_34145_40395_-	TR	TRAF
Contig1080_34145_40871_-	TR	TRAF
Contig132840_55822_61969_-	TR	Pseudo ARR-B
Contig132840_55822_61991_-	TR	Orphans
Contig132840_55822_61991_-	TR	Orphans
Contig146255_15761_18097_+	TF	bZIP
Contig146255_15796_17687_+	TF	bZIP
Contig1638_249215_257494_+	TR	Pseudo ARR-B
Contig17_261666_265876_-	TF	HB->HB-BELL
Contig17_261666_265943_-	TF	HB->HB-BELL
Contig19_53141_57163_+	TF	bHLH
Contig2073_101132_103126_+	TF	MYB->MYB-related
Contig2073_101155_103126_+	TF	MYB->MYB-related
Contig2073_101189_103126_+	TF	MYB->MYB-related
Contig2552_28356_32719_+	TF	DBP
Contig2552_30450_32719_+	TF	DBP
Contig331_497586_500841_+	TR	TAZ
Contig331_497603_500841_+	TR	TAZ
Contig331_497747_500818_+	TR	TAZ
Contig331_497747_500841_+	TR	TAZ
Contig390_172512_174723_+	TF	PLATZ
Contig4007_186044_188820_-	TR	GNAT
Contig4007_186044_188906_-	TR	GNAT
Contig4007_186044_189040_-	TR	GNAT
Contig4007_186469_188510_-	TR	GNAT
Contig4032_283069_285138_-	TF	PLATZ
Contig4032_283069_285367_-	TF	PLATZ
Contig8248_7910_9322_+	TF	C2C2->C2C2-CO-like

Supplementary Table 11. The internal scale for FPKM normalization in each sample.

**Sample**	**Total number of fragments**	**Internal scale**
CTRL_0	39,146,900	1.06369
CTRL_1	37,662,400	0.991034
ET_0	39,259,100	0.918345
ET_1	35,788,600	1.01834
JA_0	36,337,900	1.04871
JA_1	35,908,700	1.00422
LA_0	36,127,800	1.05958
LA_1	33,637,600	0.980015

Supplementary Table 12. The primer sequence for 12 target TFs using relative qRT-PCR analysis.

FUP	Forward (5' ---- 3')	Reverse (5' ---- 3')
MYB44	AGTCCATCTGGATCTGACGTC	ACCTCAGAGGAGTCGGCA
RAV1	AGCGTGTCTGGCTTGGTAC	GCTTTGGAATGGGAGTTCA
NFYA3-like	TATGGACCACAGGCCATTCT	CCCTAACCCGATTTAATGC
MYB86	GGTTGCAGAGATGTGGAAAG	CTTAGCTATCTGCGCCCATC
LBD1	CCGTATTTTCCTCCGACTGA	CAGCACAGCCATAAACAGGA
AP2-like	AAAGCTGCAAGGGCTTATGA	GCAACTCTGCCTATCCTTGC
bHLH13-like	CCCAAAGAAGGGGAAGAGTC	ATGCTTACCGGACGCAAA
SBP6	CCACGTGATTTGGGAGGCTT	ACCTGGAACAACCAATGGAC
Trihelix	TGCGAGTGAAGATGATGAGC	GCCTGTCAACACCCTCAAAT
**FDO**	**Forward (5' ---- 3')**	**Reverse (5' ---- 3')**
ICE1	TATGAGGGCTCTGGACAAC	AAACTGCGGGTCACATCATT
MYB1R1	AGGTGGCTAGTCATGCTCA	ATTGCTGAAGTGTGGCTGTG
COL	TGGGAATCGAAGTGGAGACC	CACACACGCTCGTGTCTAGA
**Housekeeping Gene**	**Forward (5' ---- 3')**	**Reverse (5' ---- 3')**
ADF4	GTGCCGATATGCTGTCTATGATTT	TCCCTCTTGAACCTGTCCTTG

References

1 Loh, S. C., Thottathil, G. P. & Othman, A. S. Identification of differentially expressed genes and signalling pathways in bark of *Hevea brasiliensis* seedlings associated with secondary laticifer differentiation using gene expression microarray. *Plant Physiology and Biochemistry* **107**, 45-55 (2016).
